# Exome sequencing identifies gene variants and networks associated with extreme respiratory outcomes following preterm birth

**DOI:** 10.1186/s12863-018-0679-7

**Published:** 2018-10-20

**Authors:** Aaron Hamvas, Rui Feng, Yingtao Bi, Fan Wang, Soumyaroop Bhattacharya, Jared Mereness, Madhurima Kaushal, C Michael Cotten, Philip L Ballard, Thomas J Mariani, PROP Investigators, Barbara Alexander, Claire Chougnet, Tari Gratton, James M Greenberg, Cathy Grisby, William Hardie, Alan H Jobe, Beth Koch, Karen McDowell, Kelly Thornton, Pamela Bates, Claudia Cleveland, Thomas Ferkol, Aaron Hamvas, Julie Hoffmann, Mark R Holland, James Kemp, Philip T Levy, Laura Linneman, Jayne Sicard-Su, Gina Simpson, Gautam K Singh, Barbara Warner, Philip L Ballard, Roberta A Ballard, David J Durand, Eric C Eichenwald, Roberta L Keller, Amir M Khan, Leslie Lusk, Jeffrey D Merrill, Dennis W Nielson, Elizabeth E Rogers, Jeanette M Asselin, Samantha Balan, Katrina Burson, Cheryl Chapin, Erna Josiah-Davis, Carmen Garcia, Hart Horneman, Rick Hinojosa, Christopher Johnson, Susan Kelley, Karin L Knowles, M Layne Lillie, Karen Martin, Sarah Martin, Julie Arldt-McAlister, Georgia E McDavid, Lori Pacello, Shawna Rodgers, Daniel K Sperry, Judy Aschner, Amy B Beller, Candice Fike, Scott Guthrie, Tina Hartert, Nathalie Maitre, Paul Moore, Mark O’ Hunt, Theresa J Rogers, Odessa L Settles, Steven Steele, Marshall Summar, Sharon Wadley, Carl D’Angio, Vasanth Kumar, Tom Mariani, Gloria Pryhuber, Clement Ren, Anne Marie Reynolds, Rita M Ryan, Kristin Scheible, Timothy Stevens, Heidie Huyck, Valerie Lunger, Shannon Castiglione, Aimee Horan, Deanna Maffet, Jane O’Donnell, Michael Sacilowski, Tanya Scalise, Elizabeth Werner, Jason Zayac, Kim Bordeaux, Pam Brown, Julia Epping, Lisa Flattery-Walsh, Donna Germuga, Nancy Jenks, Mary Platt, Eileen Popplewell, Sandra Prentice, Kim Ciccio, Michael Cotten, Kim Fisher, Jack Sharp, Judith A Voynow

**Affiliations:** 1 Department of Pediatrics, Northwestern University, Chicago, IL USA; 2 Department of Biostatistics, University of Pennsylvania, Philadelphia, PA USA; 3 Department of Preventive Medicine, Northwestern University, Chicago, IL USA; 4 Department of Pediatrics, University of Rochester, Rochester, NY USA; 5 Center for Biomedical Informatics, Washington University, St. Louis, MO USA; 6 Department of Pediatrics, Duke University, Durham, NC USA; 7 Department of Pediatrics, University of California, San Francisco, CA USA; 8 Division of Neonatology and Pediatric Molecular and Personalized Medicine Program University of Rochester Medical Center, 601 Elmwood Ave, Box 850, Rochester, NY 14642 USA; 9 Ann and Robert H. Lurie Children’s Hospital of Chicago and Northwestern University, Chicago, IL USA

**Keywords:** Prematurity and respiratory outcomes program (PROP), Bronchopulmonary dysplasia (BPD)

## Abstract

**Background:**

Previous studies have identified genetic variants associated with bronchopulmonary dysplasia (BPD) in extremely preterm infants. However, findings with genome-wide significance have been rare, and not replicated. We hypothesized that whole exome sequencing (WES) of premature subjects with extremely divergent phenotypic outcomes could facilitate the identification of genetic variants or gene networks contributing disease risk.

**Results:**

The Prematurity and Respiratory Outcomes Program (PROP) recruited a cohort of > 765 extremely preterm infants for the identification of markers of respiratory morbidity. We completed WES on 146 PROP subjects (85 affected, 61 unaffected) representing extreme phenotypes of early respiratory morbidity. We tested for association between disease status and individual common variants, screened for rare variants exclusive to either affected or unaffected subjects, and tested the combined association of variants across gene loci. Pathway analysis was performed and disease-related expression patterns were assessed. Marginal association with BPD was observed for numerous common and rare variants. We identified 345 genes with variants unique to BPD-affected preterm subjects, and 292 genes with variants unique to our unaffected preterm subjects. Of these unique variants, 28 (19 in the affected cohort and 9 in unaffected cohort) replicate a prior WES study of BPD-associated variants. Pathway analysis of sets of variants, informed by disease-related gene expression, implicated protein kinase A, MAPK and Neuregulin/epidermal growth factor receptor signaling.

**Conclusions:**

We identified novel genes and associated pathways that may play an important role in susceptibility/resilience for the development of lung disease in preterm infants.

**Electronic supplementary material:**

The online version of this article (10.1186/s12863-018-0679-7) contains supplementary material, which is available to authorized users.

## Background

Bronchopulmonary dysplasia (BPD) is the most significant pulmonary consequence of premature birth, affecting 30–60% of infants born before 28 weeks of gestation, depending on definition of the disease and the racial/ethnic population studied [1]. In contrast to most morbidities associated with extreme prematurity, the prevalence of BPD among premature infants has been stable or slightly rising over the past decade [2, 3]. Furthermore, emerging data suggests that preterm birth, and a diagnosis of BPD, is associated with increased risk for chronic lung diseases that present in older children and adults. Concordance of respiratory outcomes in monozygotic twins suggest that genetic factors may play a substantial role in influencing these outcomes [4, 5]. Genome-wide association studies (GWAS) attempting to identify BPD candidate genes using variants with a population-based frequency of more than 1% have either failed to identify candidate genes or identified genes that have not been replicated on subsequent studies [6–12].

The GWAS variant detection platform assumes that previously identified, generally common, variants with a minor allele frequency > 1% in the population, either inform directly or tag functional variants in the genome. However, a large percentage of variants in individual genomes have population-based minor allele frequencies of less than 1%, necessitating approaches that can identify rare and even unique variants. Because BPD is a complex trait that results from developmental and environmental as well as genetic factors, no single approach is likely to identify the likely multiple genes contributing to risk for BPD. Reasoning that variants that would contribute to BPD would be under negative selection pressure, Li et al. performed exome sequencing and identified extremely rare variants and associated networks of genes that were unique to the cohort with BPD [13].

The Prematurity and Respiratory Outcomes Program (PROP, http://clinicaltrials.gov/ct2/show/NCT01435187) is a geographically diverse multicenter effort across the United States to identify biochemical, physiological and genetic biomarkers that are predictive of later pulmonary status in preterm infants born at < 29 weeks of gestation [14, 15]. PROP enrolled 765 preterm infants who survived to 36-weeks of age. As an initial step in identifying genes that might be contributing to adverse short-term outcomes in this population, and because we have access to high-resolution phenotype definition in this cohort, we performed exome sequencing on a subset of infants with BPD phenotypes at the extremes of disease severity. Similar approaches have been used in small cohorts to identify candidate genes and genetic modifiers for several conditions, including cystic fibrosis, chronic obstructive pulmonary disease, macular degeneration, and preterm birth [16–20]. We hypothesized that whole exome sequencing of subjects with extreme phenotypic outcomes would be more likely to enrich for common and rare deleterious and protective genetic variants that contribute to disease risk, and we then utilized several analytic approaches with differing assumptions to identify signals that may contribute to the risk for BPD.

## Methods

*Subject and phenotype selection:* Subjects for the PROP were infants born at < 29 weeks of gestation who were followed prospectively through discharge from the NICU [1, 15]. Infants with congenital anomalies and those who were not anticipated to survive the immediate neonatal period were excluded. Saliva was collected in Oragene® kits (DNA Genotek, Inc., Ottawa, Ontario, Canada) and DNA was extracted, quantified, and stored at a central archive [15] for the PROP study. Studies at each of the participating sites were approved by the local ethics boards and all parents consented to collection and sequencing of infant DNA and reporting of de-identified results.

We defined extreme phenotypes of respiratory function at 36 weeks post menstrual age (PMA) that included; 1) the most “unaffected” group who required supplemental oxygen for < 28 days and did not require any respiratory support at 36 weeks (*n* = 61), and 2) the most “affected” group, who required mechanical ventilation for > 14 days and required continuous respiratory support through 36 weeks (*n* = 85). This respiratory support included invasive (mechanical ventilation) and non- invasive (CPAP, high flow nasal cannula > 2 l/min flow) approaches, with or without supplemental oxygen (i.e. some infants required respiratory support but with FiO2 0.21). For the purposes of this study, infants who died before 36 weeks’ PMA were excluded. The characteristics of the population are displayed in Table 1.

**Table 1:** Demographics

Infant Characteristics		Cohort total	Cases	Controls	Comparison^a^
Sample Size		146	85	61	
Gestational Age, days,		185 ± 10	180 ± 9	190 ± 8	< 0.001
Gestational Age, n (%), completed wks.					
	23	5 (3.4)	5 (5.9)	0 (0.0)	
	24	23 (15.8)	22 (25.9)	1 (1.6)	
	25	24 (16.4)	16 (18.8)	8 (13.1)	< 0.001
	26	36 (24.7)	23 (27.1)	13 (21.3)	
	27	34 (23.3)	14 (16.5)	20 (32.8)	
	28	24 (16.4)	5 (5.9)	19 (31.1)	
Female, n (%)		70 (47.9)	37 (43.5)	33 (54.1)	0.27
Race, Caucasian (%)		86 (58.9)	52 (61.2)	34 (55.7)	0.63
Multiple births, n (%)		22 (15.1)	14 (16.5)	8 (13.1)	0.75

^a^Two-group t-test (continuous) or Chi-square test (categorical)

### Sequencing and variant annotation

Exome sequencing was performed through the National Heart, Lung, and Blood Institute (NHLBI) Resequencing and Genotyping Service at the University of Washington. The quality was filtered according to GATK3.0 recommended filters; variants with any “QUAL ≤ 50.0, ABHet > 0.75, HRun > 4.0 , QD < 5.0 , SB ≥ 0.10, number of ALT ≥ 1, MQ < 40.0, or FS > 60.0” were removed. QUAL is Phred-scaled probability that support existence of ALT, ABHet is the allele balance measure for heterozygosity, HRun is the largest contiguous homopolymer run, QD is coverage-normalized quality score, SB is strand bias, MQ is RMS mapping quality, and FS is Fisher’s strand bias. The sequence data were high quality, with > 60× coverage (total sequence reads of all samples at each variant locus without filtering) and a transition to transversion ratio (Ti/Tv) of > 3, allowing the identification of 189,404 high confidence variants in the population, of which 149,344 (77.1%) had a minor allele frequency (MAF) <  0.05 (Additional file 1: Table S1). We used ANNOVAR (2015Mar22 version) to annotate all identified variants and functionally classified the exonic variants into categories including synonymous, nonsynonymous, stop gain/loss, splice site gain/loss, etc. [21]. In total, we identified 88,183 high-confidence common and rare nonsynonymous and otherwise presumed functional variants among the 146 individuals.

### Covariate determination

To derive population substructures, we used the multidimensional scaling (MDS) method implemented in PLINK (http://zzz.bwh.harvard.edu/plink/, [22]) using all variants that overlap with 1000 genome data. The first two principal components from MDS were included as covariates in subsequent association models to adjust for population stratification bias. As multiple births accounted for 22 infants (8 pairs of twins and 2 sets of triplets), we estimated pair-wise kinship coefficients using KING (Kinship-based INference for Genome-wide association studies) [23] for further inclusion as a covariance element to control for relatedness in subsequent mixed-effect models.

### Single common variant analysis

**Fig. 1:**
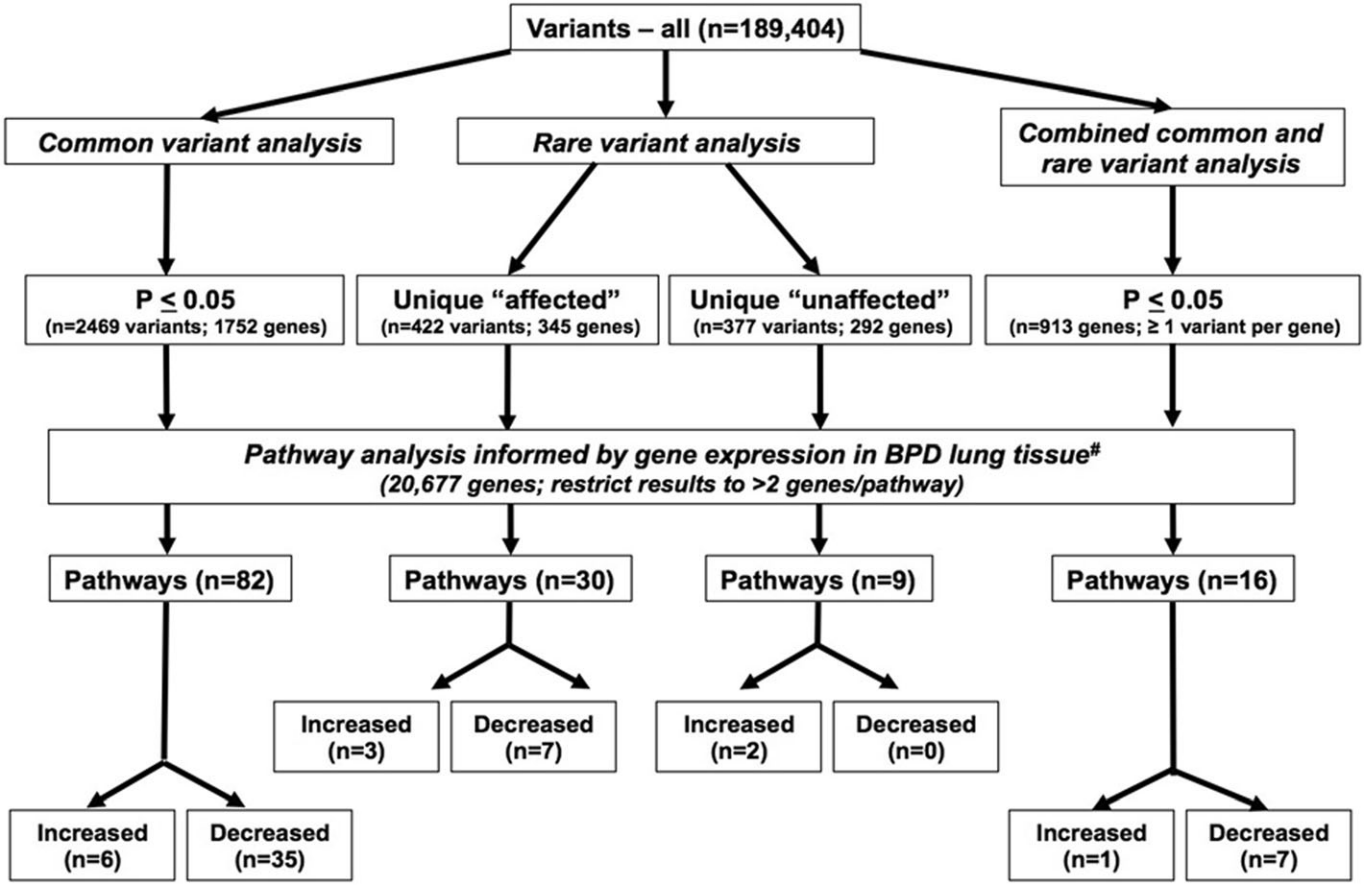
Analytical Design Schematic. Represented are the three separate approaches and the number of variants, genes and pathways identified at each step in the analysis of whole exome sequence data obtained from 146 subjects

Common variants were selected based on MAF > 0.05, missing genotype rate <  0.10, and a Hardy-Weinberg Equilibrium *p*-value > 0.01. For each common variant, we used generalized linear mixed models implemented in EMMAX (Efficient Mixed-Model Association eXpedited) to test its association with BPD, adjusting for sex, gestational age at birth, the first two principal components, and relatedness between siblings [24]. We defined significance for this first step as a *P*-value < 0.05 (Fig. 1).

### Unique variant analysis

We utilized the methods of Li, et al. [13] to identify extremely rare variants that were uniquely present in either the “affected” or “unaffected” cohorts. Briefly, we considered those variants in the cohorts that were not observed from the 1000 Genome project and dbsnp138 database, and compared them with the 1000 Genome non-synonymous variants. We used the Genomic Evolutionary Rate Profiling (GERP) score to estimate evolutionary conservation levels of these variants, where higher scores indicated greater evidence of past purifying selection [25]. To exclude stochastic mutations with low frequencies in a given population among these rare variants, we further analyzed the extremely conserved variants (GERP score > 5.83, 95% of the nonsynonymous sites in the 1000 Genome dataset) and thus considered 422 and 377 rare nonsynonymous variants identified in the 85 affected individuals and 61 unaffected individuals, respectively. When we excluded the genes that were common to both groups, we found conserved extremely rare nonsynonymous variants in 345 and 292 unique genes in the affected and unaffected cohorts, respectively (Fig. 1).

### Combined rare and common variant analysis

To test the joint effect of both common and rare variants within each gene locus, we used the Fast Family-Based Sequence Kernel Association Test (FFB-SKAT) [26]. As a powerful kernel machine method, the FFB-SKAT takes linear (additive), nonlinear, and epistasis effects into account. The analysis was adjusted for sex, gestational age at birth, population stratification as well as relatedness [27, 28]. Missing genotypes were imputed using the simple mean approach [29].

### Pathway analysis

Significantly overrepresented canonical pathways were identified using Ingenuity Pathway Analysis (IPA) software, using default settings (Qiagen). Canonical pathways with a –log(*p*-value) greater than 1.301 (*p* <  0.05) were considered significant. In addition, for directional pathway analysis, we cross-referenced these results with a previously published data set defining genome-wide expression changes in postmortem lung tissues from unrelated preterm subjects with and without BPD [30]. Briefly, probe sets for the Affymetrix HG-U133 plus array were matched using Entrez Gene IDs and NetAffx (www.netaffx.com). If the Entrez Gene ID was missing, the Official Gene Symbol was used. An expression ratio comparing BPD cases to controls was included for each gene, and if no expression value was available the gene was excluded from the analysis. Canonical pathway analysis was performed and results included z- scores denoting regulatory status (activated, inhibited) of each significant pathway if 3 or more genes were represented. Complete pathway analysis results are provided in Additional file 3: Table S3, Additional file 4: Table S4, Additional file 5: Table S5 and Additional file 7: Table S7. Pathways selected for inclusion in Fig. 3 were among the most significant, were identified in more than 1 pathway analysis results or had at least one predicted directionality, and were of biological interest.

## Results

### Common variants

**Fig. 2:**
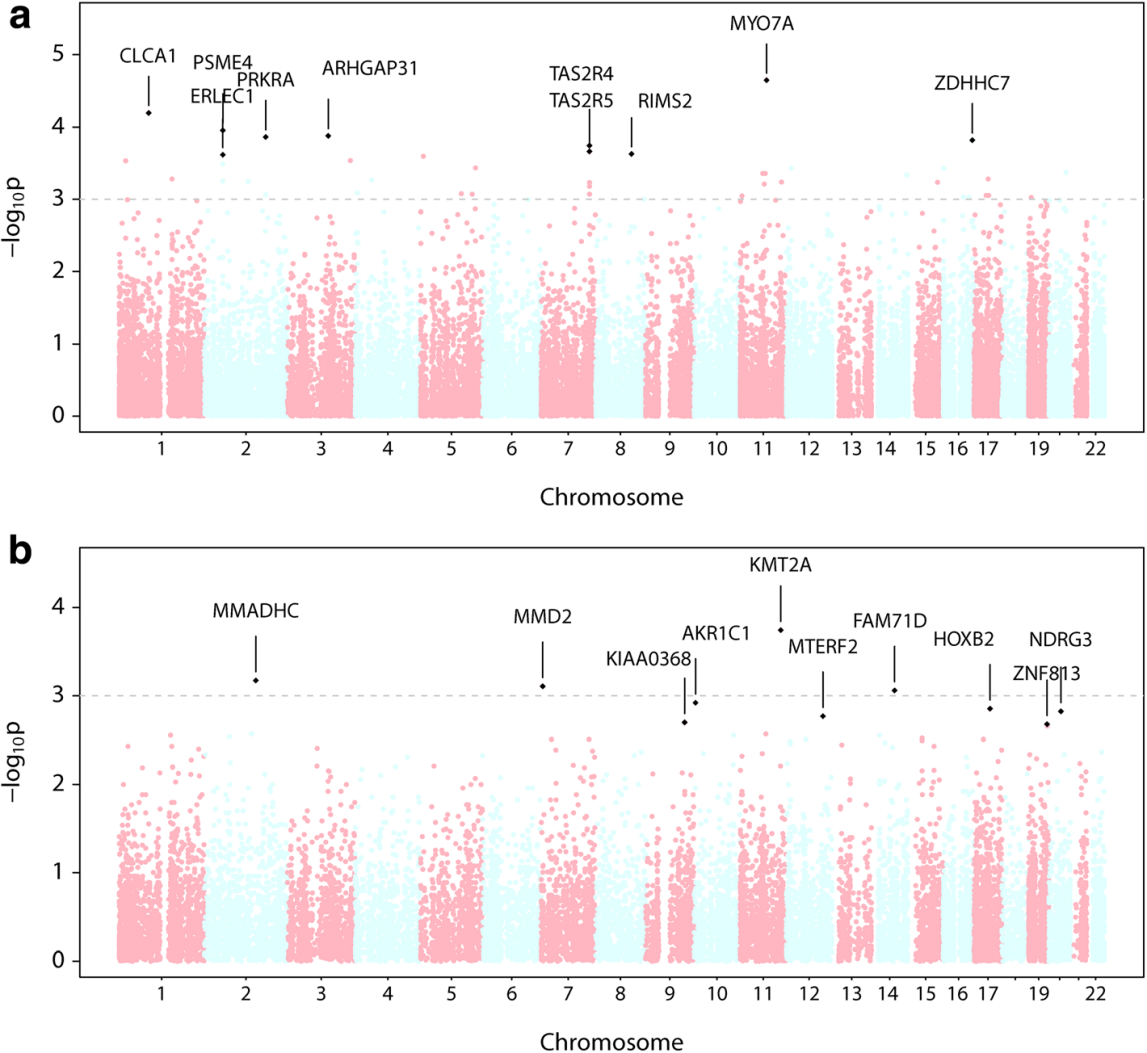
Genome-wide Association Results. We performed genome-wide association analysis using either (**a**) all common variants (MAF > 0.05) or (**b**) combined rare and common variants at each individual gene locus (FFB-SKAT), comparing the affected group to the unaffected group. Manhattan plots show the strength of association for all variants, organized by chromosomal locus, with genes demonstrating greatest association indicated by name

**Fig. 3:**
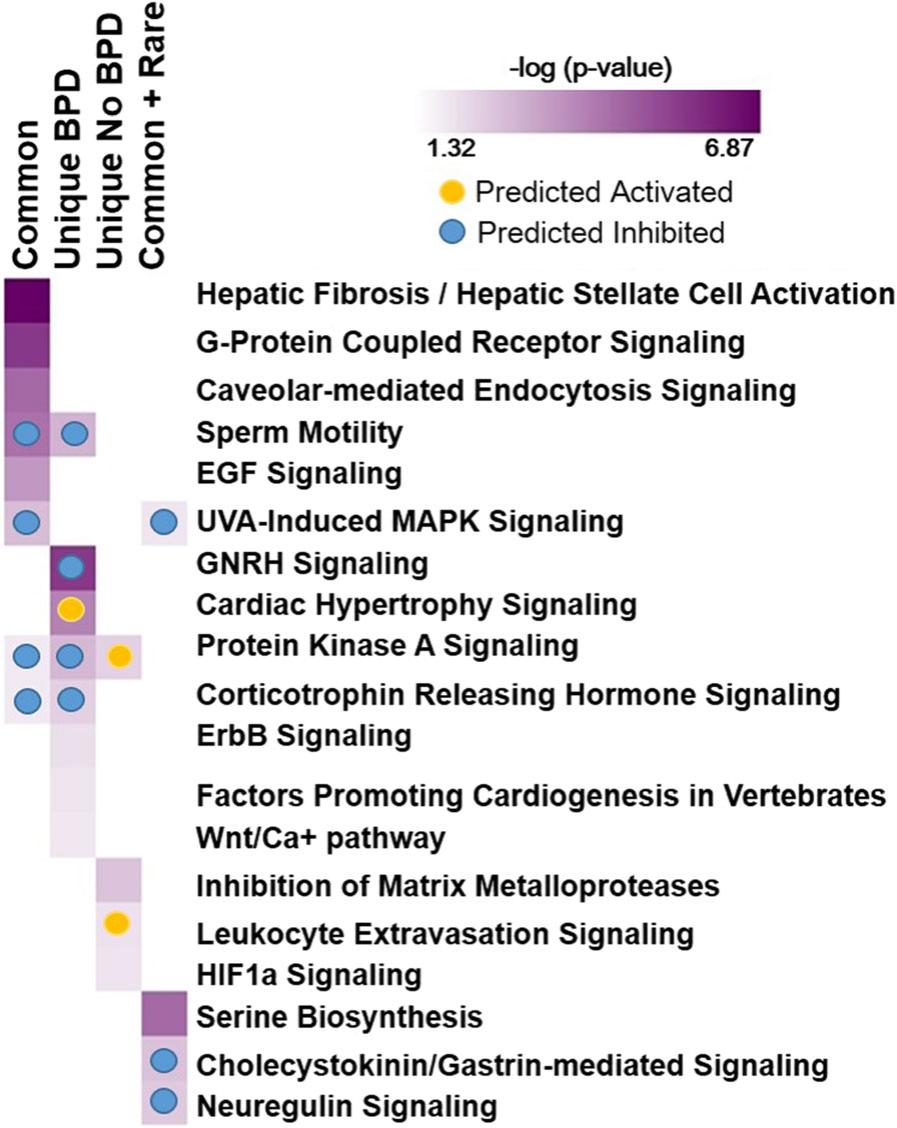
Gene Expression-Informed Pathway Analysis. Shown is a summary of pathway analysis results for genes with variants demonstrating association with respiratory outcomes. Separate analyses were performed for gene sets identified from analysis of common variants (Common), unique variants only identified in affected subjects (Unique BPD), unique variants only identified in the unaffected subjects (Unique No BPD), and combined common and rare variants (Common + Rare). Standard pathway analyses were supplemented with gene expression data from lung tissue from subjects with BPD and appropriate controls. Top canonical pathways identified (rows) are listed for each analysis (columns), with significance (− logP) and directionality (activated/inhibited). Interestingly, some biological functions were commonly identified including PKA (activated in affected subjects, inhibited in unaffected subjects), corticotropin releasing hormone (inhibited in affected), UVA-induced MAPK (inhibited in affected) and sperm motility (inhibited in affected)

We first assessed the association between common variants, as defined as a MAF > 0.05, and BPD using EMMAX while adjusting for gestational age, sex, population stratification, and relatedness between siblings (Fig. 2a and Additional file 2: Table S2). We identified a total of 2469 common variants in 1752 genes with nominal association with BPD status, but none reaching genome-wide significance. The strongest associations identified are denoted in Additional file 2: Table S2. We then used Ingenuity Pathway Analysis to interrogate the 1752 genes with common variant association *P* <  0.05 and further informed this analysis using genome-wide expression data from lung tissue of infants with BPD [30]. Among the most significant pathways identified (Additional file 4: Table S4 and Fig. 3) included Hepatic fibrosis/hepatic stellate cell activation (−log *p*-value = 6.9), G-protein receptor signaling (−log p- value = 4.1) and caveolar-mediated endocytosis signaling (−log p-value = 3.4), however BPD-specific lung tissue gene expression did not inform any directionality in these pathways (Fig. 3). Interestingly, these gene expression patterns predicted sperm motility, MAPK, protein Kinase A and corticotropin releasing hormone signaling pathways to be inhibited. Additional significant pathways identified, along with their predicted (e.g., activation/inhibition) status, are listed in Additional file 3: Table S3.

### Unique variants

Using the approach described by Li et al. [13], we identified 345 genes with extremely rare variants that were uniquely present only in our severely affected subjects, 19 of which were also among the 258 identified by Li et al. in their BPD cohort (*CDH23, CHST10, DAAM1, DYDC1, FIGN, KIAA1468, MDN1, MYOZ1, NID2, NRXN3, NUP93, PICALM, ROR1, SETBP1, SYDE2, TBC1D1, TGFBI, YLPM1, ZHX3*). Further analysis of these 345 genes identified 30 significant canonical pathways of interest (Additional file 4: Table S4 and Fig. 3). Pathways representing sperm motility, protein kinase A and corticotropin releasing hormone signaling were also detected in this analysis. In addition, this analysis predicted cardiac hypertrophy signaling to be activated and gonadotropin related hormone signaling to be inhibited.

Using this approach, we also identified a total of 292 genes with extremely rare variants that were uniquely found in our unaffected subjects (Fig. 1), 9 of which were among the 182 identified by Li et al. in their non-disease cohort (*DAG1, FBN1, GABRA2, MRPL16, NCAM1, POLR3A, PPP1R3A, SLC38A2, SOX6*). Pathway analyses using these 292 genes with variants unique to this unaffected cohort identified 9 significant canonical pathways of interest (Additional file 5: Table S5 and Fig. 3). Of particular interest, the genes with extremely rare variants unique to the unaffected subjects were associated with *activation* of protein kinase A signaling, whereas the genes in this pathway associated with BPD in the 2 preceding analyses predicted *inhibition* of protein kinase A signaling. These data may indicate a genetic basis for susceptibility and resilience from chronic lung disease in preterm infants.

### Combined rare and common variants

Hypothesizing that a combination of rare and common variants within a given gene locus are likely to contribute to risk for, or protection from, adverse respiratory outcomes, we pursued an approach to test for the combined association of all variants across individual gene loci.

Using the Fast Family-Based Sequence Kernel Association Test (FFB-SKAT) we identified 913 genes with *P* < 0.05 and 139 genes with *P* < 0.01 (Fig. 2b and Additional file 6: Table S6). Again, no genes showed evidence for significant association following adjustment for multiple testing, but the strongest associations included those for genes encoding *KMT2A* (1.8 × 10–4), *CHRDL2* (2.7 × 10–3) and *CTSK* (2.8 × 10–3). Interestingly, there were 16 genes identified that had unique variants in cases, and also identified by FFB-SKAT (*ANKRD31, ARHGAP32, C5orf66, DCLRE1A, FCAMR, KMT2A, NFX1, NRXN3, NSMAF, PIGH, PSAT1, TEX2, TGFBI, UNC45A, WDFY2, YLPM1*), and there were 12 genes identified that had unique variants in controls, and also identified by FFBSKAT (*ABCA1, ANKEF1, ANKRD17, FAM120A, MIPEP, MPZL2, PHGDH, SOX6, TPGS2, WDR36, WLS, YTHDC1*).

Pathway analysis using the 913 genes, informed by BPD-associated expression, identified 16 significant canonical pathways of interest (Additional file 7: Table S7). Similar to the common variant analysis, MAPK signaling was predicted to be inhibited in BPD. The single gene in this pathway with variants identified in both analyses was the epidermal growth factor receptor (*EGFR)*, the principal member of the ErbB receptor tyrosine kinase family, which has been implicated in alveolar formation [31]. A related pathway, the Neuregulin signaling pathway, was among the most highly significant (−log *p*-value of 1.85), and was predicted to be inhibited in BPD. Neuregulin promotes lung maturation and surfactant production, and mice deficient in neuregulin receptor (ErbB4) display BPD-like pathology [32, 33]. In this analysis, the Cholecystokinin/Gastrin-mediated Signaling pathway was also predicted to be inhibited in BPD. This is of interest given prior studies implicating gastrin-related peptide (GRP) in BPD pathogenesis [34].

For key pathways identified by our results and presented in Fig. 3, we further determined if genes with variants are expressed in human fetal lung at a time commensurate with birth at greatest risk for BPD, using data from a recently published study [35]. We found a large majority of these genes are expressed, and are therefore biologically plausible candidates for affecting disease risk/susceptibility. Specifically, we assessed the expression of all 174 genes associated with the 19 pathways. Of 62,130 genes (coding genes plus non-coding RNAs) tested for expression, 14,500 were identified as expressed (counts per million normalized counts > 5 in control/untreated condition samples). Among the 174 genes associated with these 19 pathways, 115 genes were identified as expressed in the fetal lung data. The association between the expression of genes in this data set to greater than would be expected by chance was tested using a Chi-square test (*p* < 0.001). These data, along with an assessment of their differential expression in human BPD lung tissue [30], and their expression trajectory in early human lung development [36] are presented in Additional file 8: Table S8.

## Discussion

We describe the largest exome sequencing study for respiratory outcomes of prematurity to date, and add to the body of genetic data available for this population. We focused our efforts on those subjects with extremely divergent respiratory morbidity outcomes reasoning that we would have greater power to detect highly penetrant genetic influences [16, 17, 19]. We also chose not to use conventional definitions for BPD, which are based on need for supplemental oxygen at 36 weeks post-menstrual age, since current practices for respiratory support of the premature newborn emphasize non-invasive positive pressure administration without supplemental oxygen whenever possible [1]. As would be anticipated, the cohorts were skewed with respect to gestational age, with more infants born at the earliest gestations being in the extreme affected group and those with the later gestations (though still less than 29 weeks) being more prevalent in the extreme unaffected group. Nonetheless, the common variant analysis with EMMAX and the combined common and rare variant analysis with FFB- SKAT controlled for this difference in gestational age as well as other confounders (sex and race) that could independently account for genetic differences between the two groups.

Previous candidate gene and genome-wide studies testing a million or more variants in which the MAF is > 1% have failed to identify consistent common variants, genes, or pathways that are significantly associated with respiratory morbidity following preterm birth. We report exact *p*-values, per published guidelines for statistical reporting, allowing readers to make their own judgments about appropriate corrections. Since WES allows for the identification and enumeration of both common and rare variants, we used several analytical approaches to test for those variants that are associated with our extreme 36-week respiratory outcomes [37].

Each of our approaches to identify disease risk-associated variants utilizes a different set of assumptions, which probably accounts for the differing results. To gain insight into mechanistic relevance and potential function of variants with marginal associations, we integrated functional data based on gene expression from lung tissue of infants who died of BPD [30]. Results from these analyses, particularly those including rare genetic variants, identified a small set of biological pathways that warrant additional consideration (Fig. 3).

Rare variants in 10 (of 63) genes that represent the cardiac hypertrophy signaling pathway were uniquely represented in the severely affected group. This pathway was also significantly activated in BPD based upon gene expression data. Up to 25% of infants with severe BPD may develop pulmonary hypertension, which is typically attributed to elevated pulmonary vascular resistance from arrest of pulmonary vascular development and increases the potential for mortality [38–40]. However, the presence of variants in this cardiac hypertrophy pathway suggests that there may be a subset of infants whose lung disease is complicated by cardiac or pulmonary vascular dysfunction that is genetic in origin.

Rare variants in both the gonadotropin and corticotropin releasing hormone (GnRH and CRH, respectively) pathways were also uniquely identified in the affected cohort, and were inhibited in BPD based upon gene expression (Additional file 8: Table S8). Endogenous corticosteroids increase in late gestation and enhance lung maturation, and there are previously described associations between low levels of maternal unconjugated estriol in the 2nd trimester with BPD and worse neonatal outcomes, along with multiple human and animal studies that demonstrate improved lung function and outcomes with estradiol or corticosteroid administration [41–44]. In a gene expression study of peripheral blood mononuclear cells, the GnRH pathway was activated in infants who developed BPD [45]. These observations support the possible relevance of gene variants in these two pathways to the risk for more severe respiratory disease after extremely preterm birth.

Intriguingly, rare variants among separate sets of genes in the protein kinase A (PKA) signaling pathway were uniquely identified in both the affected and unaffected groups. Twelve (of 71) individual genes in the PKA pathway had variants uniquely in the affected subjects while variants in 10 different genes in this pathway were uniquely present in unaffected subjects.

Gene expression data indicate this pathway is inhibited in subjects who develop BPD, but is activated in those without BPD. While the relevance of these observations is uncertain, PKA- cAMP signaling is known to be a critical determinant in promoting respiratory epithelial cell differentiation [37, 46, 47].

Results from a more agnostic approach, combining contributions from both common and rare variants, identified different sets of genes and pathways with potential biological significance. We noticed a convergence in many of these pathways, particularly focusing upon the EGFR family, and particularly neuregulin. Variants in 7 genes of the neuregulin signaling pathway were associated with the affected group, and this pathway was predicted to be inhibited in BPD based upon gene expression. Two additional pathways implicated variation in receptors for neuregulin in the affected group: variants in 5 genes from the EGFR superfamily- related ErbB2/ErbB3 pathway and variants in 7 genes from a MAPK pathway, where *EGFR* serves as a central hub. Gene expression data again suggested inhibition of receptor signaling in both of these cases. In vitro, neuregulin has previously been reported to stimulate surfactant production mediated by erbB2 receptor phosphorylation in alveolar type II cells [32].

Furthermore, mice deficient in the neuregulin receptor *ErbB4* [33] or *EGFR* [31] display BPD- like pathology. From these data, we speculate that genetic variants that suppress neuregulin/EGFR/ErbB4 signaling may also contribute to severity of lung disease in a subset of infants with adverse respiratory outcomes. In support of this conclusion, we found variants in multiple genes of the EGF signaling pathway were present in the affected group in both the common and combined variant analysis, and in both cases gene expression predicted this pathway was inhibited in BPD.

The lack of replication in genetic studies for BPD likely stems in large part from population differences; i.e. differences in gestational ages, limited cohort sizes, population ancestry, and, perhaps most importantly, the lack of a precise definition of BPD. The platform for variant identification plays an important role, as well. Studies using a genotyping approach with common variant-focused arrays may identify genomic regions in which specific genetic information is embedded, but as most of these variants are in non-coding regions, the data typically only infer the possibility of functional variants in the region. Conversely, sequence- based genotyping, as with exon capture sequencing used in this study, overlooks functionally relevant variants in non-coding regions.

Our studies to a certain extent have similar limitations, in that we have failed to replicate many prior observed genetic associations. One of the first genome-wide screens for BPD candidate genes identified variants in *SPOCK2*, an extracellular matrix molecule, associated with multiple, independent cohorts [8]. Another relatively large genome-wide screen identified BPD-associated variants associated with *ADARB2, CD44* and *miR-219* [6]. We are unable to replicate these associations in our population. In fact, the largest genome-wide screen to date failed to identify a single locus that met genome-wide significance [11], similar to ours.

However, and of potential greatest importance of our results, we have been able to successfully replicate the association of 28 genes with extremely rare variants previously associated with susceptibility or resistance to BPD following preterm birth [13]. In addition to these 28 genes, 4 pathways were implicated by unique variants identified in both studies (Additional file 9: Table S9 and Additional file 10: Table S10); three pathways were replicated in cases (Folate Transformations I, Factors Promoting Cardiogenesis in Vertebrates, and Sertoli Cell-Sertoli Cell Junction Signaling) and one in controls (Protein Kinase A Signaling). These data are consistent with the hypothesis that the number of potential genes related to lung maturity and defense systems is large, each with a relatively small effect size.

Our study has several additional limitations. Our definition of adverse respiratory outcome was based on short-term treatment before and at 36 weeks PMA. Definitions based on longer-term outcomes may help identify other risk variants underlying infants’ respiratory outcomes. The size of the cohort, relative to the number of variants interrogated, limits our power to identify variants with requisite statistical significance. Additional whole genome sequencing studies with larger populations will be able to address this issue of heritability with more precision. While sequencing studies combining multiple analytic approaches may help to identify risk genes and pathological mechanisms that will, in turn, permit a more precise definition of disease phenotype, only whole genome sequencing will provide the entire range of variation and permit more in-depth look at the prevalence and relevance of non-coding region variation.

## Conclusion

In summary, we used whole exome sequencing in a cohort of premature infants with the extremes of short-term respiratory outcomes, and used multiple analytical approaches to identify several genes associated with BPD. Although individual genes did not meet genome wide statistical significance, we found several candidate pathways with common themes and biological plausibility that are worthy of more in-depth exploration with additional sequencing studies.

## Additional files

Additional file 1: **Table S1.** Sequence quality summary (DOCX 24 kb)

Additional file 2: **Table S2.** Top 50 Genome-wide Common SNP Association Results (DOCX 17 kb)

Additional file 3: **Table S3.** Significant canonical pathways represented by common variants associated with BPD. (DOCX 31 kb)

Additional file 4: **Table S4.** Significant canonical pathways represented by unique variants in “affected” subjects. (DOCX 28 kb)

Additional file 5: **Table S5.** Significant canonical pathways represented by unique variants in “unaffected” subjects. (DOCX 32 kb)

Additional file 6: **Table S6.** Top 50 Genes from locus-based (FFB-SKAT) Association Analysis (DOCX 16 kb)

Additional file 7: **Table S7.** Significant canonical pathways represented by locus-based (FFB-SKAT) association results. (DOCX 14 kb)

Additional file 8: **Table S8.** Gene expression in human lung tissue. (DOCX 51 kb)

Additional file 9: **Table S9.** Significant canonical pathways represented by unique variants in “affected” subjects from Li et al......... (DOCX 14 kb)

Additional file 10: **Table S10.** Significant canonical pathways represented by unique variants in “unaffected” subjects from Li et al. (DOCX 22 kb)

## Abbreviations

BPD: Bronchopulmonary dysplasia
EMMAX: Efficient Mixed-Model Association eXpedited
FFB-SKAT: Fast Family-Based Sequence Kernel Association Test
GERP: Genomic Evolutionary Rate Profiling
GWAS: Genome-wide association studies
IPA: Ingenuity Pathway Analysis
KING: Kinship-based INference for Genome-wide association studies
MAF: Minor allele frequency
MDS: Multidimensional scaling
NHLBI: National Heart, Lung, and Blood Institute
PMA: Postmenstrual age
PROP: Prematurity and Respiratory Outcomes Program
WES: Whole Exome Sequencing

## References

[1] Poindexter BB, Feng R, Schmidt B, Aschner JL, Ballard RA, Hamvas A, Reynolds AM, Shaw PA, Jobe AH, Prematurity and Respiratory Outcomes Program (2015). Comparisons and limitations of current definitions of bronchopulmonary dysplasia for the prematurity and respiratory outcomes program. Ann Am Thorac Soc.

[2] Horbar JD, Carpenter JH, Badger GJ, Kenny MJ, Soll RF, Morrow KA, Buzas JS (2012). Mortality and neonatal morbidity among infants 501 to 1500 grams from 2000 to 2009. Pediatrics.

[3] Stoll BJ, Hansen NI, Bell EF (2015). Trends in care practices, morbidity, and mortality of extremely preterm neonates, 1993-2012. JAMA.

[4] Bhandari V, Bizzarro MJ, Shetty A, Zhong X, Page GP, Zhang H, Ment LR, Gruen JR, Group ftNGS (2006). Familial and genetic susceptibility to major neonatal morbidities in preterm twins. Pediatrics.

[5] Lavoie PM, Pham C, Jang KL (2008). Heritability of bronchopulmonary dysplasia, defined according to the consensus statement of the National Institutes of Health. Pediatrics.

[6] Ambalavanan N, Cotten CM, Page GP, Carlo WA, Murray JC, Bhattacharya S, Mariani TJ, Cuna AC, Faye-Petersen OM, Kelly D (2015). Integrated genomic analyses in bronchopulmonary dysplasia. J Pediatr.

[7] Carrera P, Di Resta C, Volonteri C, Castiglioni E, Bonfiglio S, Lazarevic D, Cittaro D, Stupka E, Ferrari M, Somaschini M (2015). Exome sequencing and pathway analysis for identification of genetic variability relevant for bronchopulmonary dysplasia (BPD) in preterm newborns: a pilot study. Clin Chim Acta.

[8] Hadchouel A, Durrmeyer X, Bouzigon E, Incitti R, Huusko J, Jarreau P-H, Lenclen R, Demenais F, Franco-Montoya M-L, Layouni I (2011). Identification of SPOCK2 as a susceptibility gene for bronchopulmonary dysplasia. Am J Respir Crit Care Med.

[9] Lal CV, Ambalavanan N (2015). Genetic predisposition to bronchopulmonary dysplasia. Semin Perinatol.

[10] Torgerson DG, Ballard P, Keller RL, Oh S, Huntsman S, Hu D, Eng C, Burchard E, Ballard R, TOLSURF Study Group. Ancestry and Genetic Associations with Bronchopulmonary Dysplasia in Preterm Infants. Am J Physiol Lung Cell Mol Physiol. 2018. 10.1152/ajplung.00073.2018 PMC6295513 30113228

[11] Wang H, St. Julien KR, Stevenson DK, Hoffmann TJ, Witte JS, Lazzeroni LC, Krasnow MA, Quaintance CC, Oehlert JW, Jelliffe-Pawlowski LL (2013). A genome-wide association study (GWAS) for bronchopulmonary dysplasia. Pediatrics.

[12] Yu KH, Li J, Snyder M, Shaw GM, O'Brodovich HM (2016). The genetic predisposition to bronchopulmonary dysplasia. Curr Opin Pediatr.

[13] Li J, Yu KH, Oehlert J, Jeliffe-Pawlowski LL, Gould JB, Stevenson DK, Snyder M, Shaw GM, O'Brodovich HM (2015). Exome sequencing of neonatal blood spots and the identification of genes implicated in bronchopulmonary dysplasia. Am J Respir Crit Care Med.

[14] Maitre N L, Ballard R A, Ellenberg J H, Davis S D, Greenberg J M, Hamvas A, Pryhuber G S (2015). Respiratory consequences of prematurity: evolution of a diagnosis and development of a comprehensive approach. Journal of Perinatology.

[15] Pryhuber GS, Maitre NL, Ballard RA, Cifelli D, Davis SD, Ellenberg JH, Greenberg JM, Kemp J, Mariani TJ, Panitch H (2015). Prematurity and respiratory outcomes program (PROP): study protocol of a prospective multicenter study of respiratory outcomes of preterm infants in the United States. BMC Pediatr.

[16] Bruse S, Moreau M, Bromberg Y, Jang JH, Wang N, Ha H, Picchi M, Lin Y, Langley RJ, Qualls C (2016). Whole exome sequencing identifies novel candidate genes that modify chronic obstructive pulmonary disease susceptibility. Hum Genomics.

[17] Emond MJ, Louie T, Emerson J, Chong JX, Mathias RA, Knowles MR, Rieder MJ, Tabor HK, Nickerson DA, Barnes KC (2015). Exome sequencing of phenotypic extremes identifies CAV2 and TMC6 as interacting modifiers of chronic Pseudomonas aeruginosa infection in cystic fibrosis. PLoS Genet.

[18] Sardell RJ, Bailey JN, Courtenay MD, Whitehead P, Laux RA, Adams LD, Fortun JA, Brantley MA, Kovach JL, Schwartz SG (2016). Whole exome sequencing of extreme age-related macular degeneration phenotypes. Mol Vis.

[19] Shtir C, Aldahmesh MA, Al-Dahmash S, Abboud E, Alkuraya H, Abouammoh MA, Nowailaty SR, Al-Thubaiti G, Naim EA, ALYounes B (2016). Exome-based case-control association study using extreme phenotype design reveals novel candidates with protective effect in diabetic retinopathy. Hum Genet.

[20] Uzun A, Schuster J, McGonnigal B, Schorl C, Dewan A, Padbury J (2016). Targeted sequencing and meta-analysis of preterm birth. PLoS One.

[21] Wang K, Li M, Hakonarson H (2010). ANNOVAR: functional annotation of genetic variants from high-throughput sequencing data. Nucleic Acids Res.

[22] Purcell S, Neale B, Todd-Brown K, Thomas L, Ferreira MA, Bender D, Maller J, Sklar P, de Bakker PI, Daly MJ (2007). PLINK: a tool set for whole-genome association and population-based linkage analyses. Am J Hum Genet.

[23] Manichaikul A, Mychaleckyj JC, Rich SS, Daly K, Sale M, Chen WM (2010). Robust relationship inference in genome-wide association studies. Bioinformatics.

[24] Zhou X, Stephens M (2012). Genome-wide efficient mixed-model analysis for association studies. Nat Genet.

[25] Davydov EV, Goode DL, Sirota M, Cooper GM, Sidow A, Batzoglou S (2010). Identifying a high fraction of the human genome to be under selective constraint using GERP++. PLoS Comput Biol.

[26] Svishcheva GR, Belonogova NM, Axenovich TI (2014). FFBSKAT: fast family-based sequence kernel association test. PLoS One.

[27] Schaid DJ, McDonnell SK, Sinnwell JP, Thibodeau SN (2013). Multiple genetic variant association testing by collapsing and kernel methods with pedigree or population structured data. Genet Epidemiol.

[28] Wu MC, Lee S, Cai T, Li Y, Boehnke M, Lin X (2011). Rare-variant association testing for sequencing data with the sequence kernel association test. Am J Hum Genet.

[29] Ionita-Laza I, Lee S, Makarov V, Buxbaum JD, Lin X (2013). Sequence kernel association tests for the combined effect of rare and common variants. Am J Hum Genet.

[30] Bhattacharya S, Go D, Krenitsky DL, Huyck HL, Solleti SK, Lunger VA, Metlay L, Srisuma S, Wert SE, Mariani TJ (2012). Genome-wide transcriptional profiling reveals connective tissue mast cell accumulation in bronchopulmonary dysplasia. Am J Respir Crit Care Med.

[31] Li J, Li Y, He H, Liu C, Li W, Xie L, Zhang Y (2016). Csk/Src/EGFR signaling regulates migration of myofibroblasts and alveolarization. Am J Physiol Lung Cell Mol Physiol.

[32] Dammann CE, Nielsen HC, Carraway KL (2003). Role of neuregulin-1 beta in the developing lung. Am J Respir Crit Care Med.

[33] Purevdorj Erkhembulgan, Zscheppang Katja, Hoymann Heinz G., Braun Armin, von Mayersbach Dietlinde, Brinkhaus Maria-Jantje, Schmiedl Andreas, Dammann Christiane E. L. (2008). ErbB4 deletion leads to changes in lung function and structure similar to bronchopulmonary dysplasia. American Journal of Physiology-Lung Cellular and Molecular Physiology.

[34] Degan S, Lopez GY, Kevill K, Sunday ME (2008). Gastrin-releasing peptide, immune responses, and lung disease. Ann N Y Acad Sci.

[35] Barrette AM, Roberts JK, Chapin C, Egan EA, Segal MR, Oses-Prieto JA, Chand S, Burlingame AL, Ballard PL (2016). Antiinflammatory effects of budesonide in human fetal lung. Am J Respir Cell Mol Biol.

[36] Kho AT, Bhattacharya S, Tantisira KG, Carey VJ, Gaedigk R, Leeder JS, Kohane IS, Weiss ST, Mariani TJ (2010). Transcriptomic analysis of human lung development. Am J Respir Crit Care Med.

[37] Gonzales LW, Guttentag SH, Wade KC, Postle AD, Ballard PL (2002). Differentiation of human pulmonary type II cells in vitro by glucocorticoid plus cAMP. Am J Physiol Lung Cell Mol Physiol.

[38] Hilgendorff A, Apitz C, Bonnet D, Hansmann G (2016). Pulmonary hypertension associated with acute or chronic lung diseases in the preterm and term neonate and infant. The European Paediatric Pulmonary Vascular Disease Network, endorsed by ISHLT and DGPK. Heart.

[39] Khemani E, McElhinney DB, Rhein L, Andrade O, Lacro RV, Thomas KC, Mullen MP (2007). Pulmonary artery hypertension in formerly premature infants with bronchopulmonary dysplasia: clinical features and outcomes in the surfactant era. Pediatrics.

[40] Kim DH, Kim HS, Choi CW, Kim EK, Kim BI, Choi JH (2012). Risk factors for pulmonary artery hypertension in preterm infants with moderate or severe bronchopulmonary dysplasia. Neonatology.

[41] Doyle LW, Halliday HL, Ehrenkranz RA, Davis PG, Sinclair JC (2014). An update on the impact of postnatal systemic corticosteroids on mortality and cerebral palsy in preterm infants: effect modification by risk of bronchopulmonary dysplasia. J Pediatr.

[42] Jelliffe-Pawlowski LL, Shaw GM, Stevenson DK, Oehlert JW, Quaintance C, Santos AJ, Baer RJ, Currier RJ, O’Brodovich HM, Gould JB (2012). Risk of bronchopulmonary dysplasia by second trimester maternal serum levels of alpha-fetoprotein, human chorionic gonadotrophin, and unconjugated Estriol. Pediatr Res.

[43] McCurnin DC, Pierce RA, Willis BC, Chang LY, Yoder BA, Yuhanna IS, Ballard PL, Clyman RI, Waleh N, Maniscalco W (2009). Postnatal estradiol up-regulates lung nitric oxide synthases and improves lung function in bronchopulmonary dysplasia. Am J Respir Crit Care Med.

[44] Nykänen P, Anttila E, Heinonen K, Hallman M, Voutilainen R (2007). Early hypoadrenalism in premature infants at risk for bronchopulmonary dysplasia or death. Acta Paediatr.

[45] Pietrzyk JJ, Kwinta P, Wollen EJ, Bik-Multanowski M, Madetko-Talowska A, Günther C-C, Jagła M, Tomasik T, Saugstad OD (2013). Gene expression profiling in preterm infants: new aspects of bronchopulmonary dysplasia development. PLoS One.

[46] Ballard PL, Gonzales LW, Williams MC, Roberts JM, Jacobs MM (1991). Differentiation of type II cells during explant culture of human fetal lung is accelerated by endogenous prostanoids and adenosine 3′,5′-monophosphate. Endocrinology.

[47] Wang Y, Maciejewski BS, Lee N, Silbert O, McKnight NL, Frangos JA, Sanchez-Esteban J (2006). Strain-induced fetal type II epithelial cell differentiation is mediated via cAMP-PKA-dependent signaling pathway. Am J Physiol Lung Cell Mol Physiol.

