# Supplementary material for: Exome sequencing identifies gene variants and networks associated with extreme respiratory outcomes following preterm birth
Source: BMC Genet. 2018 Oct 20;19:94. doi: 10.1186/s12863-018-0679-7 (PMC6195962; doi:10.1186/s12863-018-0679-7)
Supplement: Supplementary file 1 — Table S1. Sequence quality summary (DOCX 24 kb) [file 12863_2018_679_MOESM1_ESM.docx]

Supplemental Table 1. Sequence quality summary

**Quality Measure Mean ± Std. Dev.**

Per variant

Depth 59 ± 34

Genotype quality (GQ) 85.0 ± 23.4

Coverage 9485 ± 5446

Allele balance for heterozygosity 0.51 ± 0.11 Largest contiguous homopolymer run 0.49 ± 0.78 Quality normalized by coverage 14.0 ± 4.76

RMS mapping quality 58.2 ± 2.4

Fisher's strand bias 6.5 ± 8.6

Per subjects

Missing genotypes 1476 ± 409

Average Coverage 60 ± 8.9

Average Genotype Quality 85.0 ± 2.4

Homozygous variants 177467 ± 2018

Heterozygous variants 14669 ± 2046

TS, transitions 16898 ±1638

TV, transversions 5341 ± 490

Ti/Tv (TS/TV) Ratio 3.16 ± 0.04
