# Supplementary material for: Exome sequencing identifies gene variants and networks associated with extreme respiratory outcomes following preterm birth
Source: BMC Genet. 2018 Oct 20;19:94. doi: 10.1186/s12863-018-0679-7 (PMC6195962; doi:10.1186/s12863-018-0679-7)
Supplement: Supplementary file 2 — Table S2. Top 50 Genome-wide Common SNP Association Results (DOCX 17 kb) [file 12863_2018_679_MOESM2_ESM.docx]

Supplemental Table 2. Top 50 Genome-wide Common SNP Association Results

| **CHR** | **POS** | **SNP** | **ALT** | **REF** | **OR** | **p-value** | **Gene** | **Function** |
| --- | --- | --- | --- | --- | --- | --- | --- | --- |
| 11 | 76919468 | rs1320703 | A | T | 1.27 | 0.00002 | MYO7A | intron |
| 1 | 86947975 | rs1321694 | T | A | 0.76 | 0.00006 | CLCA1 | coding-synonymous |
| 2 | 54133744 | rs805316 | C | T | 0.73 | 0.00011 | PSME4 | coding-synonymous |
| 3 | 119133554 | rs61740281 | A | G | 1.36 | 0.00013 | ARHGAP31 | coding-synonymous |
| 2 | 179300979 | rs77419724 | T | A | 1.43 | 0.00014 | PRKRA | missense |
| 16 | 85009970 | rs7195377 | A | G | 1.33 | 0.00015 | ZDHHC7 | coding-synonymous |
| 7 | 141478800 | rs2234002 | A | G | 1.28 | 0.00018 | TAS2R4 | missense |
| 7 | 141490238 | rs2227264 | T | G | 1.27 | 0.00022 | TAS2R5 | missense |
| 8 | 105261725 | rs2028945 | A | G | 1.39 | 0.00024 | RIMS2 ERLEC1,GPR75- | coding-synonymous |
| 2 | 54041983 | rs2542571 | T | G | 0.80 | 0.00024 | ASB3 | intron |
| 5 | 7802363 | rs2290910 | T | C | 1.29 | 0.00025 | ADCY2 | coding-synonymous |
| 3 | 183861243 | rs843358 | G | A | 1.26 | 0.00029 | EIF2B5 | missense |
| 1 | 19611227 | rs1065658 | A | G | 0.71 | 0.00029 | AKR7A3 | coding-synonymous |
| 2 | 54115904 | rs805400 | G | A | 0.78 | 0.00032 | PSME4 | coding-synonymous |
| 5 | 160097496 | rs958912 | A | G | 1.34 | 0.00037 | ATP10B | missense |
| 16 | 961051 | rs2277893 | T | C | 1.28 | 0.00037 | LMF1 | coding-synonymous |
| 12 | 14587301 | rs3213764 | G | A | 1.24 | 0.00037 | ATF7IP | missense |
| 20 | 50235577 | rs2295004 | A | G | 1.41 | 0.00043 | ATP9A | coding-synonymous |
| 11 | 73715542 | rs2075577 | G | A | 1.24 | 0.00044 | UCP3 | coding-synonymous |
| 11 | 65685225 | rs7947504 | T | C | 1.42 | 0.00044 | C11orf68 | missense |
| 14 | 103429420 | rs34301396 | C | T | 1.46 | 0.00045 | CDC42BPB | coding-synonymous |
| 1 | 155149718 | rs3814316 | A | G | 1.25 | 0.00052 | TRIM46 | coding-synonymous |
| 17 | 41004503 | rs33916389 | A | G | 0.63 | 0.00053 | AOC3 | coding-synonymous |
| 4 | 47945295 | rs28642966 | T | C | 1.32 | 0.00054 | CNGA1 | missense |
| 2 | 54120025 | rs805408 | T | A | 0.78 | 0.00056 | PSME4 | missense |
| 2 | 128601490 | rs11688522 | T | C | 1.21 | 0.00057 | none | intergenic |
| 11 | 120823608 | rs2298725 | A | G | 1.26 | 0.00058 | GRIK4 | coding-synonymous |
| 15 | 86287910 | rs11073517 | T | C | 1.26 | 0.00058 | AKAP13 | utr-3 |
| 7 | 141537735 | rs12669721 | G | T | 1.25 | 0.00059 | PRSS37 | missense |
| 11 | 70118489 | rs546502 | A | G | 1.29 | 0.00061 | PPFIA1 | missense |
| 7 | 141464765 | rs2270009 | T | C | 1.25 | 0.00066 | TAS2R3 | coding-synonymous |
| 7 | 141478308 | rs2233998 | C | T | 1.25 | 0.00066 | TAS2R4 | missense |
| 4 | 6304087 | rs1046316 | A | G | 1.24 | 0.00082 | WFS1 | coding-synonymous |
| 5 | 118484804 | rs7734532 | G | A | 0.82 | 0.00083 | DMXL1 | coding-synonymous |
| 5 | 150565008 | rs248427 | T | C | 1.21 | 0.00084 | CCDC69 | missense |
| 7 | 141478574 | rs2234001 | C | G | 1.25 | 0.00085 | TAS2R4 | missense |
| 2 | 179300971 | rs62176107 | A | G | 1.34 | 0.00086 | PRKRA | coding-synonymous |
| 17 | 42961009 | rs2289677 | C | T | 1.24 | 0.00088 | EFTUD2 | intron |
| 17 | 34871813 | rs752493 | C | T | 1.43 | 0.00088 | MYO19 | coding-synonymous |
| 11 | 4928866 | rs7941509 | T | C | 0.80 | 0.00090 | OR51A7 | coding-synonymous |
| 16 | 58587737 | rs11866002 | T | C | 1.23 | 0.00093 | CNOT1 | coding-synonymous |

| 16 | 75269534 | rs3743613 | T | C | 1.21 | 0.00094 | BCAR1 | coding-synonymous |
| --- | --- | --- | --- | --- | --- | --- | --- | --- |
| 19 | 8161450 | rs3829817 | T | C | 1.27 | 0.00094 | FBN3 | missense |
| 8 | 142489410 | rs2748418 | G | A | 1.24 | 0.00100 | FLJ43860 | missense |
| 6 | 133072650 | rs1883617 | G | A | 1.21 | 0.00101 | VNN2 | coding-synonymous |
| 1 | 24932223 | rs62623442 | C | G | 1.47 | 0.00101 | NCMAP | missense |
| 1 | 24932232 | rs74062045 | A | G | 1.47 | 0.00101 | NCMAP | coding-synonymous |
| 20 | 31876681 | rs1078761 | G | A | 1.23 | 0.00102 | BPIFB1 | missense |
| 11 | 103780455 | rs10791649 | G | A | 1.21 | 0.00103 | PDGFD | coding-synonymous |
| 20 | 1115919 | rs2235587 | G | A | 1.38 | 0.00104 | PSMF1 | missense |
