# Supplementary material for: Exome sequencing identifies gene variants and networks associated with extreme respiratory outcomes following preterm birth
Source: BMC Genet. 2018 Oct 20;19:94. doi: 10.1186/s12863-018-0679-7 (PMC6195962; doi:10.1186/s12863-018-0679-7)
Supplement: Supplementary file 3 — Table S3. Significant canonical pathways represented by common variants associated with BPD. (DOCX 31 kb) [file 12863_2018_679_MOESM3_ESM.docx]

Supplemental Table 3. Significant canonical pathways represented by common variants associated with BPD.

| **Canonical Pathways** | **-log**  **(p-value)** | **z- score** | **Molecules** |
| --- | --- | --- | --- |
| Hepatic Fibrosis / | 6.87 | DNP | MYH6,FN1,ICAM1,LEPR,EGF,COL4A2,MYH11,MYH7B,COL |
| Hepatic Stellate Cell Activation |  |  | 17A1,COL15A1,COL5A1,COL16A1,NGFR,LAMA1,COL22A1  ,LBP,COL18A1,EGFR,COL4A1,COL2A1,MYH7,COL1A1,CO |
|  |  |  | L5A3,COL13A1,COL6A3,CD40,COL23A1,MYH3,COL24A1, |
| G-Protein Coupled | 4.13 | DNP | COL4A4,PDGFD,MMP9 ENPP6,PTK2B,MC3R,PTGER3,VIPR2,HTR1D,RAPGEF4,H |
| Receptor Signaling |  |  | RH3,SHC1,PLCB1,GRM6,PIK3R2,AVPR1B,ADRB2,PRKCA, PIK3C2B,ADCY2,GNAS,PIK3C2A,IKBKE,DRD2,FPR1,PDE1 |
|  |  |  | C,ADCY9,GABBR2,VIPR1,NPR3,RAP1GAP,GNAO1,CALCR |
| Caveolar-mediated | 3.36 | DNP | ,PRKAG2,DUSP4,ADORA2A  ARCN1,HLA-A,EGF,ITGB8,ITGAL,ITGAE,ITGA3,HLA- |
| Endocytosis Signaling Sperm Motility | 3.24 | -0.83 | C,INSR,ITGA7,ITGB5,PRKCA,EGFR ATP1A4,GNAS,PRKCQ,PTK2B,PLA2R1,PLA2G1B,CACNA1 |
|  |  |  | H,PLA2G3,ITPR1,CNGA1,PLA2G7,PDE1C,CNGB1,ITPR3,P RKAG2,PLCB1,CATSPER1,PRKCA |
| Actin Cytoskeleton | 2.94 | -0.45 | RAC2,FGD3,MYH6,FN1,EGF,MYLK,MYH11,MYH7B,BCAR1 |
| Signaling |  |  | ,SHC1,ITGA3,IQGAP2,CYFIP2,PIK3R2,LBP,SSH2,ACTN1,P IK3C2B,PIK3C2A,CSK,MYH7,TTN,DOCK1,PIP5K1C,MYH3, |
| Axonal Guidance | 2.89 | DNP | PDGFD RAC2,ADAM17,BMP4,EPHB2,EGF,SEMA4F,BCAR1,ADAM |
| Signaling |  |  | TS2,EIF4E,SHC1,EPHB1,ITGA3,EPHA8,ADAM28,NGFR,PL |
|  |  |  | CB1,PLXNB1,ARHGEF11,ABLIM2,PIK3R2,SEMA3B,PTCH2  ,PRKCA,SEMA3E,PIK3C2B,TUBB1,EPHB4,GNAS,STK36,P |
|  |  |  | RKCQ,KALRN,FES,PIK3C2A,SLIT2,PLXND1,EFNA1,DOCK 1,WIPF1,GNAO1,PRKAG2,GNAT2,PDGFD,EPHA2,MMP9 |
| Virus Entry via | 2.89 | DNP | PIK3C2B,RAC2,ITGA3,PRKCQ,PIK3C2A,HLA-C,HLA- |
| Endocytic Pathways Leukocyte | 2.80 | 0.85 | A,CLTCL1,PIK3R2,ITGB8,CXADR,ITGAL,ITGB5,PRKCA PIK3C2B,RAC2,ICAM1,PRKCQ,PIK3C2A,PTK2B,MMP14,M |
| Extravasation Signaling |  |  | MP15,RAPGEF4,ITGAL,BCAR1,SELPLG,TEC,WIPF1,ITGA3  ,RAP1GAP,ICAM3,MAPK10,PIK3R2,CTTN,MMP9,MMP17,A |
|  |  |  | CTN1,PRKCA |
| Paxillin Signaling | 2.79 | -1.27 | PIK3C2B,PIK3C2A,PTK2B,CSK,ITGB8,BCAR1,ITGAL,ITGA E,DOCK1,ITGA3,MAPK10,PIK3R2,ITGA7,ACTN1,ITGB5 |
| NF-κB Activation by Viruses | 2.72 | DNP | PIK3C2B,ITGA3,PRKCQ,PIK3C2A,CD4,IKBKE,IKBKAP,PIK 3R2,ITGAL,ITGB5,PRKCA,CR2 |
| EGF Signaling | 2.62 | 0.00 | SHC1,PIK3C2B,JAK1,PIK3C2A,ITPR3,EGF,PIK3R2,ITPR1, PRKCA,EGFR |
| Endothelin-1 Signaling | 2.56 | -1.09 | PIK3C2B,ADCY2,PRKCQ,GNAS,PIK3C2A,GUCY2D,PTGS1 |
|  |  |  | ,PLA2R1,PLA2G1B,PLA2G3,ITPR1,PLA2G7,SHC1,ADCY9, GNAO1,ITPR3,MAPK10,PLCB1,GNAT2,PIK3R2,PRKCA |
| cAMP-mediated signaling | 2.53 | -0.82 | ENPP6,ADCY2,GNAS,VIPR2,MC3R,PTGER3,HTR1D,RAPG EF4,DRD2,CNGA1,HRH3,FPR1,PDE1C,AKAP13,ADCY9,G |
|  |  |  | ABBR2,CNGB1,VIPR1,NPR3,RAP1GAP,GNAO1,GRM6,DU |
| Cellular Effects of | 2.51 | DNP | SP4,ADORA2A,ADRB2 SLC4A5,MYH6,ADCY2,GNAS,GUCY2D,CACNA1C,MYH7,M |
| Sildenafil (Viagra) |  |  | YLK,MYH11,ITPR1,MYH7B,PDE1C,ADCY9,ITPR3,MYH3,P RKAG2,PLCB1 |

| CXCR4 Signaling | 2.48 | -1.21 | PIK3C2B,ADCY2,GNAS,PRKCQ,PIK3C2A,CD4,DIRAS3,ITP R1,BCAR1,ADCY9,DOCK1,GNAO1,ITPR3,MAPK10,PLCB1, |
| --- | --- | --- | --- |
|  |  |  | GNAT2,ARHGEF11,PIK3R2,PRKCA |
| Atherosclerosis Signaling | 2.31 | DNP | SELE,ICAM1,APOF,PLA2R1,COL2A1,PLA2G1B,PLA2G3,PL A2G7,SELPLG,COL1A1,COL5A3,SELP,CD40,COL18A1,PD |
|  |  |  | GFD,MMP9 |
| Activation of IRF by Cytosolic Pattern | 2.29 | 0.63 | IFIH1,TRAF3,CD40,DDX58,ZBP1,MAPK10,IKBKE,STAT2,IK BKAP,ISG15 |
| Recognition Receptors Oxidative Ethanol | 2.26 | DNP | ALDH3A2,ALDH1A2,ACSS2,ALDH3A1,ALDH9A1 |
| Degradation III Ethanol Degradation II | 2.26 | DNP | ALDH3A2,ALDH1A2,ACSS2,PECR,ALDH3A1,ALDH9A1,AD |
|  |  |  | H4 |
| CD40 Signaling | 2.14 | -0.33 | PIK3C2B,TRAF3,ICAM1,CD40,PIK3C2A,PTGS1,MAPK10,IK BKE,IKBKAP,PIK3R2 |
| Non-Small Cell Lung Cancer Signaling | 2.14 | -0.33 | PIK3C2B,RXRG,FHIT,PIK3C2A,ITPR3,EGF,PIK3R2,ITPR1, PRKCA,EGFR |
| Antigen Presentation | 2.12 | DNP | HLA-G,NLRC5,HLA-A,HLA-C,HLA-DQA1,CIITA,MR1 |
| Pathway  HER-2 Signaling in | 2.11 | DNP | PIK3C2B,TSC1,PRKCQ,PIK3C2A,TSC2,EGF,PIK3R2,ITGB8 |
| Breast Cancer  iCOS-iCOSL Signaling | 2.10 | -0.63 | ,ITGB5,PRKCA,EGFR PIK3C2B,PRKCQ,PIK3C2A,CD4,CSK,HLA- |
| in T Helper Cells |  |  | DQA1,IKBKE,HLA- |
| Dendritic Cell | 2.09 | 0.89 | DQB1,ITPR1,INPP5D,SHC1,CD40,ITPR3,PIK3R2 PIK3C2B,ICAM1,PIK3C2A,LEPR,FCGR2A,HLA-A,HLA- |
| Maturation |  |  | DQA1,COL2A1,IKBKE,HLA- DQB1,COL1A1,COL5A3,CD40,HLA- |
|  |  |  | C,NGFR,MAPK10,PLCB1,STAT2,PIK3R2,COL18A1 |
| FAK Signaling | 2.08 | DNP | DOCK1,PIK3C2B,ITGA3,CAPN11,PIK3C2A,CSK,HMMR,EG F,CAPN2,PIK3R2,BCAR1,EGFR |
| Intrinsic Prothrombin Activation Pathway | 2.08 | 0.00 | COL1A1,KLKB1,COL5A3,F5,COL2A1,COL18A1 |
| Renin-Angiotensin Signaling | 2.07 | -1.60 | PIK3C2B,ADCY2,GNAS,PRKCQ,PIK3C2A,PTK2B,ITPR1,SH C1,ADCY9,ITPR3,PRKAG2,MAPK10,PIK3R2,PRKCA |
| Regulation of Cellular | 2.05 | -0.82 | ITGA3,CNGB1,CAPN11,EGF,CAPN2,CAST,CNGA1,ACTN1, |
| Mechanics by Calpain Protease |  |  | EGFR |
| GABA Receptor Signaling | 2.05 | 0.71 | GABRG3,GABBR2,ADCY9,ADCY2,GABRG2,GNAS,GABRG 1,UBQLN1,GABRD,ALDH9A1 |
| UVA-Induced MAPK | 2.04 | -0.58 | PIK3C2B,PIK3C2A,PARP4,MAPK10,PLCB1,PIK3R2,RPS6K |
| Signaling  Ephrin A Signaling | 2.02 | DNP | A2,SMPD3,PARP9,PRKCA,PARP1,EGFR PIK3C2B,EPHA8,PIK3C2A,NGFR,PIK3R2,BCAR1,EPHA2,E |
| GDNF Family Ligand- | 2.01 | -0.71 | FNA1 SHC1,PIK3C2B,PSPN,ARTN,PIK3C2A,DOK2,ITPR3,MAPK1 |
| Receptor Interactions |  |  | 0,PIK3R2,ITPR1 |
| Nitric Oxide Signaling in the Cardiovascular | 2.00 | -1.73 | PIK3C2B,PRKCQ,PIK3C2A,GUCY2D,RYR2,CACNA1C,ITP R1,PDE1C,BDKRB2,ITPR3,PRKAG2,PIK3R2,PRKCA |
| System  Integrin Signaling | 1.94 | 0.43 | PIK3C2B,RAC2,PIK3C2A,CAPN11,DIRAS3,MYLK,ITGB8,B |
|  |  |  | CAR3,ITGAL,TTN,BCAR1,ITGAE,SHC1,DOCK1,WIPF1,ITG |
| The Visual Cycle | 1.91 | DNP | A3,CAPN2,PIK3R2,ITGA7,CTTN,ACTN1,ITGB5 DHRS3,LRAT,RDH8,RPE65 |
| Ethanol Degradation IV | 1.89 | DNP | ALDH3A2,ALDH1A2,ACSS2,ALDH3A1,ALDH9A1 |

| GPCR-Mediated Integration of | 1.88 | DNP | ADCY9,ADCY2,GNAS,VIPR1,GLP2R,ITPR3,PRKAG2,PLCB 1,ITPR1,ADRB2 |
| --- | --- | --- | --- |
| Enteroendocrine |  |  |  |
| Signaling Exemplified by an L Cell |  |  |  |
| FcγRIIB Signaling in B | 1.88 | -1.13 | SHC1,PIK3C2B,CD79B,PIK3C2A,MAPK10,PIK3R2,INPP5D |
| Lymphocytes Aldosterone Signaling | 1.81 | -2.11 | PIK3C2B,NEDD4,PRKCQ,PIK3C2A,DNAJC6,DNAJC1,ITPR |
| in Epithelial Cells |  |  | 1,DNAJB13,HSPA8,SACS,PIP5K1C,ITPR3,PLCB1,NR3C2,C RYAA/LOC102724652,PIK3R2,PRKCA |
| Retinol Biosynthesis | 1.80 | DNP | AADAC,DHRS3,LRAT,CES1,RDH8,PNLIPRP3 |
| Synaptic Long Term | 1.76 | -1.00 | GNAS,PRKCQ,GUCY2D,RYR2,PLA2R1,PLA2G1B,PLA2G3, |
| Depression |  |  | ITPR1,PLA2G7,ITPR3,GNAO1,PLCB1,GRM6,GNAT2,PPP2 |
| eNOS Signaling | 1.76 | -1.16 | R5C,PRKCA PIK3C2B,ADCY2,GNAS,PRKCQ,PIK3C2A,ITPR1,CNGA1,B |
|  |  |  | DKRB2,HSPA8,ADCY9,CNGB1,ITPR3,PRKAG2,PIK3R2,BD KRB1,PRKCA |
| UVB-Induced MAPK | 1.76 | -0.71 | PIK3C2B,PRKCQ,PIK3C2A,MAPK10,PIK3R2,EIF4E,PRKCA |
| Signaling Phototransduction | 1.76 | DNP | ,EGFR GUCA2B,CNGB1,GUCY2D,PRKAG2,GNAT2,SAG,OPN4,C |
| Pathway  Bupropion Degradation | 1.74 | DNP | NGA1  CYP4F8,CYP4B1,CYP2A6 (includes |
|  |  |  | others),CYP2C8,CYP2S1 |
| Gap Junction Signaling | 1.74 | DNP | PIK3C2B,TUBB1,ADCY2,GNAS,PRKCQ,PIK3C2A,GUCY2D, EGF,ITPR1,DRD2,ADCY9,ITPR3,PRKAG2,PLCB1,PIK3R2, |
| Calcium-induced T | 1.74 | -0.38 | EGFR,PRKCA  PRKCQ,CD4,ITPR3,HLA-DQA1,NR4A1,CAPN2,HLA- |
| Lymphocyte Apoptosis |  |  | DQB1,ITPR1,PRKCA |
| Gαi Signaling | 1.72 | 0.54 | SHC1,GABBR2,ADCY9,ADCY2,GNAS,NPR3,RAP1GAP,PT GER3,PRKAG2,GRM6,HTR1D,DRD2,HRH3,FPR1 |
| IL-4 Signaling | 1.72 | DNP | SYNJ2,SHC1,PIK3C2B,JAK1,PIK3C2A,HLA- DQA1,NR3C2,HLA-DQB1,PIK3R2,INPP5D |
| Insulin Receptor Signaling | 1.72 | -0.26 | PIK3C2B,TSC1,JAK1,PIK3C2A,EIF2B3,EIF4E,INPP5D,PTP RF,SYNJ2,SHC1,TSC2,EIF2B5,PRKAG2,INSR,PIK3R2 |
| Histamine Degradation | 1.71 | DNP | ALDH3A2,ALDH1A2,ALDH3A1,ALDH9A1 |
| Gαs Signaling | 1.71 | -1.51 | ADCY9,ADCY2,CNGB1,GNAS,VIPR1,MC3R,VIPR2,RYR2,P RKAG2,RAPGEF4,ADORA2A,CNGA1,ADRB2 |
| phagosome formation | 1.71 | DNP | PIK3C2B,ITGA3,PRKCQ,FN1,TLR5,PIK3C2A,FCGR2A,DIR AS3,PLA2R1,PLCB1,PIK3R2,INPP5D,PRKCA |
| Gustation Pathway | 1.70 | DNP | ENPP6,ADCY2,TAS2R50,GNAS,TAS2R7,ITPR1,TAS2R3,P |
| Natural Killer Cell | 1.68 | DNP | DE1C,TAS2R4,ADCY9,ITPR3,TAS2R5,PRKAG2,P2RX7 SYNJ2,SHC1,RAC2,NCR1,PIK3C2B,PRKCQ,PIK3C2A,FCG |
| Signaling  G Beta Gamma | 1.65 | -0.30 | R2A,SH3BP2,PIK3R2,INPP5D,CD300A,PRKCA SHC1,ADCY2,GNAS,PRKCQ,CAV2,GNAO1,PRKAG2,GNAT |
| Signaling |  |  | 2,KCNJ6,PRKCA,EGFR |
| CREB Signaling in Neurons | 1.65 | -1.81 | PIK3C2B,ADCY2,GNAS,PRKCQ,PIK3C2A,ITPR1,ADCY9,S HC1,POLR2A,GRIK4,ITPR3,GNAO1,PRKAG2,PLCB1,GRM |
| ILK Signaling | 1.61 | -0.47 | 6,GNAT2,PIK3R2,PRKCA PIK3C2B,MYH6,FN1,PIK3C2A,DIRAS3,MUC1,MYH7,ITGB8, |
|  |  |  | MYH11,MYH7B,DOCK1,MAPK10,MYH3,PPP2R5C,PIK3R2, |
| Acetone Degradation I | 1.60 | DNP | ACTN1,MMP9,ITGB5,NACA CYP4F8,CYP4B1,CYP2A6 (includes |
| (to Methylglyoxal) |  |  | others),CYP2C8,CYP2S1 |

| Macropinocytosis Signaling | 1.58 | -1.34 | PIK3C2B,PRKCQ,PIK3C2A,EGF,PIK3R2,ITGB8,PDGFD,ITG B5,PRKCA |
| --- | --- | --- | --- |
| Ephrin Receptor | 1.58 | 0.00 | RAC2,GRIN3B,EPHB4,GNAS,KALRN,EPHB2,EGF,BCAR1, |
| Signaling |  |  | EFNA1,SHC1,WIPF1,ITGA3,EPHB1,EPHA8,GNAO1,GNAT2  ,PDGFD,EPHA2 |
| Autoimmune Thyroid | 1.57 | DNP | HLA-G,CD40,HLA-A,HLA-C,HLA-DQA1,HLA-DQB1,TG |
| Disease Signaling Complement System | 1.57 | DNP | MASP2,C7,C1QA,C6,CR2,C5 |
| Estrogen Biosynthesis | 1.52 | DNP | CYP4F8,CYP4B1,HSD17B12,CYP2A6 (includes others),CYP2C8,CYP2S1 |
| Noradrenaline and Adrenaline | 1.52 | DNP | ALDH3A2,ALDH1A2,PECR,ALDH3A1,ALDH9A1,ADH4 |
| Degradation |  |  |  |
| Thrombin Signaling | 1.50 | -0.24 | PIK3C2B,ADCY2,GNAS,PRKCQ,PIK3C2A,DIRAS3,EGF,MY LK,ITPR1,ADCY9,SHC1,GNAO1,ITPR3,PLCB1,GNAT2,ARH |
| Fatty Acid α-oxidation | 1.47 | DNP | GEF11,PIK3R2,EGFR,PRKCA ALDH3A2,ALDH1A2,ALDH3A1,ALDH9A1 |
| Inhibition of Matrix | 1.47 | DNP | ADAM17,MMP14,MMP15,THBS2,MMP17,MMP9 |
| Metalloproteases PTEN Signaling | 1.46 | -0.58 | SYNJ2,MAST2,SHC1,RAC2,ITGA3,NGFR,IKBKE,INSR,PIK3 |
| GPCR-Mediated | 1.42 | DNP | R2,CNKSR3,BCAR1,INPP5D,EGFR ADCY9,ADCY2,GNAS,PRKCQ,ITPR3,PRKAG2,PLCB1,ITP |
| Nutrient Sensing in |  |  | R1,RAPGEF4,PRKCA |
| Enteroendocrine Cells Putrescine | 1.40 | DNP | ALDH3A2,ALDH1A2,ALDH3A1,ALDH9A1 |
| Degradation III Role of JAK1 and | 1.40 | DNP | IL7R,SHC1,PIK3C2B,JAK1,FES,PTK2B,PIK3C2A,PIK3R2 |
| JAK3 in γc Cytokine |  |  |  |
| Signaling Sphingosine-1- | 1.38 | -0.91 | PIK3C2B,ADCY9,ADCY2,GNAS,PTK2B,PIK3C2A,DIRAS3,P |
| phosphate Signaling Role of NFAT in | 1.38 | -0.83 | LCB1,PIK3R2,PDGFD,SMPD3,ASAH1 PIK3C2B,GNAS,PRKCQ,PIK3C2A,CD79B,FCGR2A,CD4,HL |
| Regulation of the Immune Response |  |  | A-DQA1,IKBKE,ITPR1,HLA- DQB1,GNAO1,ITPR3,PLCB1,GNAT2,IKBKAP,PIK3R2 |
| Mechanisms of Viral | 1.38 | DNP | SH3GL1,NEDD4,PRKCQ,PDCD6IP,VPS37C,PRKCA |
| Exit from Host Cells Leptin Signaling in | 1.37 | -2.00 | PIK3C2B,ADCY9,ADCY2,GNAS,PIK3C2A,LEPR,PRKAG2,P |
| Obesity Gαq Signaling | 1.35 | -0.78 | LCB1,PIK3R2 PIK3C2B,GNAS,PRKCQ,PIK3C2A,PTK2B,CSK,DIRAS3,IKB |
|  |  |  | KE,ITPR1,ITPR3,CALCR,PLCB1,PIK3R2,AVPR1B,PRKCA |
| Protein Kinase A Signaling | 1.33 | -0.58 | ENPP6,PTK2B,MYLK,PTPN5,PTPRF,CDC25B,NGFR,PLCB 1,PTPRZ1,PTCH2,PRKCA,ADCY2,PTPRE,GNAS,PRKCQ,P |
|  |  |  | TPRD,RYR2,PTPN3,ITPR1,TCF3,TTN,CNGA1,PDE1C,ADC Y9,AKAP13,PTPRU,PYGM,CNGB1,PTPRB,ITPR3,PRKAG2, |
|  |  |  | DUSP4,EYA1 |
| α-Adrenergic Signaling | 1.33 | -0.63 | ADCY9,ADCY2,GNAS,PRKCQ,PYGM,ITPR3,PRKAG2,SLC 8A2,ITPR1,PRKCA |
| Corticotropin Releasing Hormone Signaling | 1.33 | -0.63 | ADCY9,ADCY2,GNAS,PRKCQ,GUCY2D,GNAO1,ITPR3,NR 4A1,PRKAG2,ITPR1,PTCH2,PRKCA |
| CDK5 Signaling | 1.33 | 0.00 | LAMA5,ADCY9,ADCY2,ITGA3,GNAS,NGFR,LAMA1,LAMB1, |
|  |  |  | MAPK10,PRKAG2,PPP2R5C |

DNP=Direction Not Predicted
