# Supplementary material for: Exome sequencing identifies gene variants and networks associated with extreme respiratory outcomes following preterm birth
Source: BMC Genet. 2018 Oct 20;19:94. doi: 10.1186/s12863-018-0679-7 (PMC6195962; doi:10.1186/s12863-018-0679-7)
Supplement: Supplementary file 4 — Table S4. Significant canonical pathways represented by unique variants in “affected” subjects. (DOCX 28 kb) [file 12863_2018_679_MOESM4_ESM.docx]

Supplemental Table 4. Significant canonical pathways represented by unique variants in “affected” subjects.

| **Canonical Pathways** | **-log(p- value)** | **z- score** | **Molecules** |
| --- | --- | --- | --- |
| GNRH Signaling | 4.12 | -1.667 | MAP3K9,PAK1,MAP3K13,CREBBP,DNM3,ITPR1,MAP3K |
| Cardiac Hypertrophy | 2.96 | 1 | 3,PRKD1,GNRHR MAP3K9,EIF2B4,PLCE1,MAP3K13,CREBBP,IGF1R,EIF2 |
| Signaling ERK5 Signaling | 2.76 | -0.447 | B1,MEF2A,MEF2C,MAP3K3 CREBBP,MEF2A,MEF2C,WNK1,MAP3K3 |
| Synaptic Long Term | 2.45 | 0.378 | PRKG1,PLCE1,GRID2,IGF1R,ITPR1,PLA2G12B,PRKD1 |
| Depression  D-myo-inositol-5- | 2.40 | DNP | NUDT5,MTMR4,PLCE1,PTPN2,PPM1H,PTPRM,PTPN22 |
| phosphate Metabolism Sperm Motility | 2.18 | -0.816 | PRKG1,PLCE1,ITPR1,MST1R,PLA2G12B,PRKD1 |
| Protein Kinase A Signaling  D-myo-inositol (1,4,5,6)- | 2.12  2.05 | -0.302  DNP | PHKB,PLCE1,PTPN2,PTPN9,PTPRB,CREBBP,ITPR1,PT PRM,PTPN21,PTPN22,PRKD1,AKAP11 NUDT5,MTMR4,PTPN2,PPM1H,PTPRM,PTPN22 |
| Tetrakisphosphate |  |  |  |
| Biosynthesis  D-myo-inositol (3,4,5,6)- | 2.05 | DNP | NUDT5,MTMR4,PTPN2,PPM1H,PTPRM,PTPN22 |
| tetrakisphosphate |  |  |  |
| Biosynthesis Xenobiotic Metabolism | 1.87 | DNP | MAP3K9,MAP3K13,CREBBP,CHST10,NDST2,FMO5,EIF2 |
| Signaling HGF Signaling | 1.82 | -0.447 | AK3,MAP3K3,PRKD1 MAP3K9,PAK1,MAP3K13,MAP3K3,PRKD1 |
| Agrin Interactions at | 1.82 | DNP | NRG1,PAK1,ERBB4,LAMA2 |
| Neuromuscular Junction 3-phosphoinositide | 1.80 | DNP | NUDT5,MTMR4,PTPN2,PPM1H,PTPRM,PTPN22 |
| Degradation Superpathway of | 1.75 | DNP | NUDT5,MTMR4,PLCE1,PTPN2,PPM1H,PTPRM,PTPN22 |
| Inositol Phosphate |  |  |  |
| Compounds Corticotropin Releasing | 1.73 | -1 | CREBBP,MEF2A,MEF2C,ITPR1,PRKD1 |
| Hormone Signaling 3-phosphoinositide | 1.67 | DNP | NUDT5,MTMR4,PTPN2,PPM1H,PTPRM,PTPN22 |
| Biosynthesis  Germ Cell-Sertoli Cell | 1.63 | DNP | MAP3K9,PAK1,MAP3K13,A2M,MAP3K3,CTNND1 |
| Junction Signaling ErbB Signaling | 1.51 | DNP | NRG1,PAK1,ERBB4,PRKD1 |
| HIPPO signaling | 1.51 | DNP | SMAD2,FAT4,MST1,PARD3 |
| CD27 Signaling in | 1.46 | DNP | MAP3K9,MAP3K13,MAP3K3 |
| Lymphocytes PPARα/RXRα Activation | 1.43 | 0.447 | SMAD2,PLCE1,ACOX1,CREBBP,MEF2C,ACVR2A |
| AMPK Signaling | 1.43 | -1 | PPM1B,TBC1D1,CREBBP,ARID2,ACACA,PHF10 |
| Sertoli Cell-Sertoli Cell | 1.43 | DNP | MAP3K9,PRKG1,MAP3K13,MAGI2,A2M,MAP3K3 |
| Junction Signaling Role of NFAT in Cardiac | 1.42 | 0 | PLCE1,IGF1R,MEF2A,MEF2C,ITPR1,PRKD1 |
| Hypertrophy |  |  |  |

Unfolded protein response Factors Promoting Cardiogenesis in Vertebrates

1.42 DNP DNAJC3, EIF2AK3, EIF2A

1.42 DNP SMAD2, MEF2C, ACVR2A, PRKD1

Wnt/Ca+ pathway 1.38 DNP PLCE1, CREBBP, ROR1

Phospholipase C Signaling

1.33 -1 PLCE1, CREBBP, MEF2A, MEF2C, ITPR1, PLA2G12B, PRKD1

RAR Activation 1.32 DNP SMAD2, TRIM24, CREBBP, ARID2, PRKD1, PHF10

DNP=Direction Not Predicted
