# Supplementary material for: Exome sequencing identifies gene variants and networks associated with extreme respiratory outcomes following preterm birth
Source: BMC Genet. 2018 Oct 20;19:94. doi: 10.1186/s12863-018-0679-7 (PMC6195962; doi:10.1186/s12863-018-0679-7)
Supplement: Supplementary file 5 — Table S5. Significant canonical pathways represented by unique variants in “unaffected” subjects. (DOCX 32 kb) [file 12863_2018_679_MOESM5_ESM.docx]

Supplemental Table 5. Significant canonical pathways represented by unique variants in “unaffected” subjects.

**Canonical Pathways -log(p- value)**

**z- score**

**Molecules**

Inhibition of Matrix

Metalloproteases 1.94 DNP MMP16,MMP13,MMP2

TGFBR2,PTPRG,ADD3,CNGA4,PTPRD,PTPN13,S MAD4,PPP1R3A,PTPRT,EP300 TAF6L,RUNX2,TRRAP,PPARGC1A,EP300 TGFBR2,RUNX2,SMAD4,EP300

| Protein Kinase A Signaling | 1.71 | 1 |
| --- | --- | --- |
| Estrogen Receptor Signaling | 1.71 | DNP |
| TGF-β Signaling | 1.67 | 0 |

Regulation of Cellular Mechanics by Calpain Protease

| 1.5 | DNP | CNGA4,TLN1,CAPN2 |
| --- | --- | --- |
| 1.48 | 1.633 | WIPF1,MMP16,CTNNA3,MMP13,MMP2,MAP3K4 |
| 1.45 | DNP | MMP16,MMP13,MMP2,EP300 |

Leukocyte Extravasation Signaling HIF1α Signaling

Antiproliferative Role of TOB in T

Cell Signaling 1.41 DNP TGFBR2,SMAD4

Gαs Signaling 1.36 DNP ADD3,CNGA4,LHCGR,EP300

DNP=Direction Not Predicted
