# Supplementary material for: Exome sequencing identifies gene variants and networks associated with extreme respiratory outcomes following preterm birth
Source: BMC Genet. 2018 Oct 20;19:94. doi: 10.1186/s12863-018-0679-7 (PMC6195962; doi:10.1186/s12863-018-0679-7)
Supplement: Supplementary file 6 — Table S6. Top 50 Genes from locus-based (FFB-SKAT) Association Analysis (DOCX 16 kb) [file 12863_2018_679_MOESM6_ESM.docx]

Supplemental Table 6. Top 50 Genes from locus-based (FFB-SKAT) Association Analysis

|  | **Number of common/rare** |  |  | Transcription | Transcription |
| --- | --- | --- | --- | --- | --- |
| **Gene** | **SNPs** | **P-value** | **Chromosome** | **Start** | **End** |
| KMT2A | 3/14 | 1.8E-04 | 11 | 118307204 | 118397539 |
| MMADHC | 1/9 | 6.7E-04 | 2 | 150426146 | 150444330 |
| MMD2 | 1/7 | 7.8E-04 | 7 | 4945619 | 4998844 |
| FAM71D | 3/9 | 8.7E-04 | 14 | 67656109 | 67695267 |
| AKR1C1 | 0/7 | 1.2E-03 | 10 | 5005453 | 5020158 |
| HOXB2 | 0/8 | 1.4E-03 | 17 | 46620018 | 46622393 |
| NDRG3 | 0/5 | 1.5E-03 | 20 | 35280168 | 35374541 |
| MTERF2 | 1/6 | 1.7E-03 | 12 | 107371068 | 107380944 |
| KIAA0368 | 4/18 | 2.0E-03 | 9 | 114122972 | 114247025 |
| ZNF813 | 7/11 | 2.1E-03 | 19 | 53970988 | 53997546 |
| NDUFA3 | 1/2 | 2.2E-03 | 19 | 54606159 | 54610281 |
| CHRDL2 | 3/13 | 2.7E-03 | 11 | 74407472 | 74442430 |
| THSD7B | 8/16 | 2.7E-03 | 2 | 137748461 | 138435287 |
| SLC22A17 | 0/7 | 2.8E-03 | 14 | 23815519 | 23821660 |
| CTSK | 0/2 | 2.8E-03 | 1 | 150768683 | 150780917 |
| ABLIM1 | 4/15 | 2.8E-03 | 10 | 116190868 | 116444414 |
| NAT8 | 4/8 | 2.9E-03 | 2 | 73867849 | 73869537 |
| IVD | 1/7 | 3.0E-03 | 15 | 40697685 | 40713512 |
| ZNRF2 | 0/1 | 3.1E-03 | 7 | 30323922 | 30407308 |
| DHRS13 | 2/4 | 3.1E-03 | 17 | 27224798 | 27230089 |
| PRIM1 | 3/1 | 3.1E-03 | 12 | 57125363 | 57146146 |
| NDUFB2 | 0/3 | 3.1E-03 | 7 | 140396480 | 140406446 |
| RPUSD2 | 1/7 | 3.2E-03 | 15 | 40861491 | 40866893 |
| ETV6 | 1/5 | 3.3E-03 | 12 | 11802787 | 12048325 |
| GEMIN2 | 0/5 | 3.4E-03 | 14 | 39583487 | 39606177 |
| RNU6-19P | 1/4 | 3.5E-03 | 12 | 11944823 | 12079107 |
| CDX2 | 1/4 | 3.6E-03 | 13 | 28536204 | 28543505 |
| LDLRAP1 | 1/4 | 3.7E-03 | 1 | 25870075 | 25895377 |
| MIR631 | 1/0 | 3.7E-03 | 15 | 75645951 | 75646026 |
| AQP10 | 3/3 | 3.7E-03 | 1 | 154293591 | 154297801 |
| SLC38A6 | 2/4 | 3.8E-03 | 14 | 61447831 | 61550451 |
| VGLL3 | 0/2 | 3.9E-03 | 3 | 86987122 | 87040257 |
| PBDC1 | 0/1 | 3.9E-03 | X | 75392770 | 75398033 |
| SIPA1L2 | 7/27 | 4.0E-03 | 1 | 232533711 | 232651243 |
| HECW1 | 6/24 | 4.1E-03 | 7 | 43152197 | 43602938 |
| RAB40B | 0/5 | 4.1E-03 | 17 | 80614942 | 80656598 |
| KCNH2 | 5/5 | 4.2E-03 | 7 | 150642043 | 150675402 |
| PHF20L1 | 1/13 | 4.3E-03 | 8 | 133787603 | 133861052 |
| CYB5R3 | 2/6 | 4.3E-03 | 22 | 43013845 | 43042995 |
| TRMT112 | 0/1 | 4.5E-03 | 11 | 64083963 | 64085556 |
| MPP7 | 2/4 | 4.5E-03 | 10 | 28339922 | 28571067 |

| NFIC | 1/2 | 4.6E-03 | 19 | 3359560 | 3469215 |
| --- | --- | --- | --- | --- | --- |
| ZNF334 | 1/22 | 4.6E-03 | 20 | 45128268 | 45142198 |
| ADI1 | 1/3 | 4.6E-03 | 2 | 3501689 | 3523350 |
| DUSP22 | 2/5 | 4.7E-03 | 6 | 292056 | 351355 |
| MIR2053 | 1/0 | 4.7E-03 | 8 | 113655721 | 113655812 |
| OR2G3 | 5/8 | 4.8E-03 | 1 | 247768887 | 247769817 |
| OR51I1 | 3/16 | 4.8E-03 | 11 | 5461771 | 5462783 |
| NOM1 | 5/14 | 4.9E-03 | 7 | 156742416 | 156765876 |
| PRSS48 | 6/2 | 4.9E-03 | 4 | 152198324 | 152212605 |
