# Supplementary material for: Exome sequencing identifies gene variants and networks associated with extreme respiratory outcomes following preterm birth
Source: BMC Genet. 2018 Oct 20;19:94. doi: 10.1186/s12863-018-0679-7 (PMC6195962; doi:10.1186/s12863-018-0679-7)
Supplement: Supplementary file 7 — Table S7. Significant canonical pathways represented by locus-based (FFB-SKAT) association results. (DOCX 14 kb) [file 12863_2018_679_MOESM7_ESM.docx]

Supplemental Table 7. Significant canonical pathways represented by locus-based (FFB-SKAT) association results.

| **Canonical Pathways** | **-log(p- value)** | **z- score** | **Molecules** |
| --- | --- | --- | --- |
| Serine Biosynthesis | 3.35 | DNP | PSAT1,DUSP26,PHGDH |
| Superpathway of Serine and Glycine Biosynthesis I | 2.83 | DNP | PSAT1,DUSP26,PHGDH |
| Retinoic acid Mediated | 2.21 | 0.00 | TIPARP,ART1,DAP3,TNFSF10,IFNA21,PARP1 |
| Apoptosis Signaling Cholecystokinin/Gastrin- | 1.97 | -0.33 | 1,CRABP2 ROCK1,DIRAS3,HRAS,IL37,FNBP1,IL36B,EGF |
| mediated Signaling Neuregulin Signaling | 1.85 | -1.89 | R,PRKCB,ATF2 NRG3,PIK3R1,HRAS,TMEFF2,ERBB2,ITGA4,E |
|  |  |  | GFR,PRKCB |
| phagosome formation | 1.77 | DNP | TLR2,MRC1,DIRAS3,PIK3R1,FCGR2B,FNBP1, FCAMR,ITGA4,PRKCB |
| Death Receptor Signaling | 1.74 | -1.41 | ROCK1,DIABLO,CASP6,TIPARP,ART1,TNFSF 10,PARP11,HTRA2 |
| p53 Signaling | 1.60 | -0.82 | CASP6,STAG1,PIK3R1,CSNK1D,PIDD1,CCND 1,BIRC5,CHEK1 |
| Estrogen-Dependent Breast | 1.59 | DNP | IGF1,PIK3R1,HRAS,CCND1,EGFR,ATF2 |
| Cancer Signaling IL-8 Signaling | 1.45 | -1.16 | ROCK1,NAPEPLD,DIRAS3,PIK3R1,GPLD1,HR |
|  |  |  | AS,LIMK2,CXCL1,CCND1,FNBP1,EGFR,PRKC B |
| Bladder Cancer Signaling | 1.42 | DNP | MMP26,HRAS,FGF3,ERBB2,CCND1,MMP17,E |
| Pancreatic Adenocarcinoma | 1.42 | -1.13 | GFR NAPEPLD,PIK3R1,GPLD1,ERBB2,STAT1,CCN |
| Signaling  Huntington's Disease Signaling | 1.41 | DNP | D1,BIRC5,EGFR GLS,PIK3R1,NAPG,HRAS,RCOR1,AP2A2,ATF |
|  |  |  | 2,CASP6,PSME1,IGF1,HAP1,RASA1,PRKCB,E |
| Endometrial Cancer Signaling | 1.41 | DNP | GFR PIK3R1,ILK,HRAS,ERBB2,CCND1 |
| UVA-Induced MAPK Signaling | 1.40 | -0.38 | TIPARP,PIK3R1,ART1,HRAS,PARP11,STAT1,E GFR |
| Colorectal Cancer Metastasis | 1.32 | 1.39 | WNT10B,ADRBK1,WNT9B,PIK3R1,DIRAS3,HR |
| Signaling |  |  | AS,BIRC5,CCND1,TLR2,MMP26,STAT1,MMP1 7,FNBP1,EGFR |

DNP=Direction Not Predicted
