# Supplementary material for: Exome sequencing identifies gene variants and networks associated with extreme respiratory outcomes following preterm birth
Source: BMC Genet. 2018 Oct 20;19:94. doi: 10.1186/s12863-018-0679-7 (PMC6195962; doi:10.1186/s12863-018-0679-7)
Supplement: Supplementary file 8 — Table S8. Gene expression in human lung tissue. (DOCX 51 kb) [file 12863_2018_679_MOESM8_ESM.docx]

Supplemental Table 8. Gene expression in human lung tissue.

**Genes**

**Expression in Expression in**

**Human Fetal Lung BPD Lung Tissue**

**Expression change with growth in**

**Normal Lung Development**

Caveolar-mediated Endocytosis Signaling

| ARCN1 EGF EGFR HLA-A HLA-C INSR ITGA3 ITGA7 ITGAE ITGAL ITGB5 ITGB8  PRKCA | D D D D D D D D D ND D ND  D | NS NS I NS NS NS NS NS NS NS NS I  NS | I  I  I  I I NS I NS R NS R R  I |
| --- | --- | --- | --- |
| Cardiac Hypertrophy Signaling | | | |
| CREBBP | D | NS | NS |
| EIF2B1 | D | NS | NS |
| EIF2B4 | D | NS | R |
| IGF1R | D | NS | NS |
| MAP3K13 | D | NS | I |
| MAP3K3 | D | NS | I |
| MAP3K9 | D | NS | I |
| MEF2A | D | NS | I |
| MEF2C | D | NS | I |
| PLCE1 | D | NS | NS |
| Factors Promoting Cardiogenesis in Vertebrates | | | |
| ACVR2A | D | NS | I |
| MEF2C | D | NS | I |
| PRKD1 | D | NS | I |
| SMAD2 | D | NS | I |
| Cholecystokinin/Gastrin-mediated Signaling | | | |
| ATF2 | D | NS | NS |
| DIRAS3 | ND | NS | NS |
| EGFR | D | I | I |
| FNBP1 | D | NS | NS |
| HRAS | D | NS | R |
| IL36B | ND | NS | NS |
| IL37 | ND | NS | NS |
| PRKCB | D | NS | R |
| ROCK1 | D | NS | I |

Corticotropin Releasing Hormone Signaling

ADCY2 ND NS R

| ADCY9 CREBBP GNAO1 GNAS GUCY2D ITPR1 ITPR3 MEF2A MEF2C NR4A1 PRKAG2 PRKCA PRKCQ PRKD1  PTCH2 | D D ND D ND D D D D D D D D D  ND | NS NS R NS NS NS NS NS NS NS NS NS NS NS  NS | I NS R NS NS I  I NS I  I  I  I  I  I  R |
| --- | --- | --- | --- |
|  |  | EGF Signaling |  |
| EGF | D | NS | I |
| EGFR | D | I | I |
| ITPR1 | D | NS | I |
| ITPR3 | D | NS | I |
| JAK1 | D | NS | I |
| PIK3C2A | D | NS | NS |
| PIK3C2B | D | NS | I |
| PIK3R2 | ND | NS | NS |
| PRKCA | D | NS | I |
| SHC1 | D | NS | R |
|  |  | ErbB Signaling |  |
| ERBB4 | ND | NS | NS |
| NRG1 | ND | NS | I |
| PAK1 | D | NS | R |
| PRKD1 | D | NS | I |
|  |  | GNRH Signaling |  |
| CREBBP | D | NS | NS |
| DNM3 | ND | NS | I |
| GNRHR | ND | NS | I |
| ITPR1 | D | NS | I |
| MAP3K13 | D | NS | I |
| MAP3K3 | D | NS | I |
| MAP3K9 | D | NS | I |
| PAK1 | D | NS | R |
| PRKD1 | D | NS | I |
| G-Protein Coupled Receptor Signaling | | | |
| ADCY2 | ND | NS | R |
| ADCY9 | D | NS | I |
| ADORA2A | ND | NS | I |
| ADRB2 | D | R | I |

| AVPR1B CALCR DRD2 DUSP4 ENPP6 FPR1 GABBR2 GNAO1 GNAS GRM6 HRH3 HTR1D IKBKE MC3R NPR3 PDE1C PIK3C2A PIK3C2B PIK3R2 PLCB1 PRKAG2 PRKCA PTGER3 PTK2B RAP1GAP RAPGEF4 SHC1 VIPR1  VIPR2 | ND ND ND D ND ND ND ND D ND ND ND D ND ND ND ND ND ND D D D ND D D D D ND  ND | NS NS NS NS NS NS NS R NS NS NS NS NS NS NS NS NS NS NS R NS NS NS NS NS NS NS NS  R | I  I NS I  I  R R R NS NS R NS NS NS I  I NS I NS R  I  I  I  I  I  I  R  I  I |
| --- | --- | --- | --- |
| CD40 COL13A1 COL15A1 COL16A1 COL17A1 COL18A1 COL1A1 COL22A1 COL23A1 COL24A1 COL2A1 COL4A1 COL4A2 COL4A4 COL5A1  COL5A3 | Hepatic F  D ND D D ND D D ND ND D D D D D D  D | ibrosis / Hepatic Stellate Cel  NS NS NS NS NS I NS NS NS NS NS NS NS NS NS  NS | l Activation  NS R NS R  I NS I  R  I  I  R  I  I  I  I  I |

| COL6A3 EGF EGFR FN1 ICAM1 LAMA1 LBP LEPR MMP9 MYH11 MYH3 MYH6 MYH7 MYH7B NGFR  PDGFD | D D D D D D ND D ND D ND ND ND ND ND  D | NS NS I NS NS NS NS NS NS NS NS NS NS NS NS  NS | I  II  R I R I  I NS NS NS R R NS R  I |
| --- | --- | --- | --- |
|  |  | HIF1α Signaling |  |
| EP300 | D | NS | NS |
| MMP13 | D | NS | I |
| MMP16 | D | NS | NS |
| MMP2 | D | NS | I |
| Inhibition of Matrix Metalloproteases | | | |
| MMP13 | D | NS | NS |
| MMP16 | D | NS | R |
| MMP2 | D | NS | I |
| Leukocyte Extravasation Signaling | | | |
| CTNNA3 | ND | NS | R |
| MAP3K4 | D | NS | NS |
| MMP13 | D | NS | NS |
| MMP16 | D | NS | R |
| MMP2 | D | NS | I |
| WIPF1 | D | I | R |
|  |  | Neuregulin Signaling |  |
| EGFR | D | I | I |
| ERBB2 | D | R | R |
| HRAS | D | NS | R |
| ITGA4 | D | NS | I |
| NRG3 | ND | NS | I |
| PIK3R1 | D | NS | NS |
| PRKCB | D | NS | R |
| TMEFF2 | ND | NS | I |
| Protein Kinase A Signaling | | | |
| ADCY2 | ND | NS | R |
| ADCY9 | D | NS | I |
| ADD3 | D | NS | NS |
| AKAP11 | D | NS | I |

| AKAP13 | D | NS | I |
| --- | --- | --- | --- |
| CDC25B | D | I | R |
| CNGA1 | ND | NS | I |
| CNGA4 | ND | NS | I |
| CNGB1 | ND | NS | NS |
| CREBBP | D | NS | NS |
| DUSP4 | D | NS | I |
| ENPP6 | ND | NS | I |
| EP300 | D | NS | NS |
| EYA1 | ND | NS | R |
| GNAS | D | NS | NS |
| ITPR1 | D | NS | I |
| ITPR3 | D | NS | I |
| MYLK | D | NS | I |
| NGFR | ND | NS | R |
| PDE1C | D | NS | I |
| PHKB | D | NS | R |
| PLCB1 | D | R | R |
| PLCE1 | D | NS | NS |
| PPP1R3A | ND | NS | NS |
| PRKAG2 | D | NS | I |
| PRKCA | D | NS | I |
| PRKCQ | D | NS | I |
| PRKD1 | D | NS | I |
| PTCH2 | ND | NS | R |
| PTK2B | D | NS | I |
| PTPN13 | D | NS | I |
| PTPN2 | D | NS | I |
| PTPN21 | D | R | R |
| PTPN22 | ND | NS | I |
| PTPN3 | D | NS | I |
| PTPN5 | ND | NS | I |
| PTPN9 | D | NS | I |
| PTPRB | D | R | R |
| PTPRD | D | NS | I |
| PTPRE | D | NS | NS |
| PTPRF | D | NS | R |
| PTPRG | D | NS | R |
| PTPRM | D | R | I |
| PTPRT | ND | NS | I |
| PTPRU | D | NS | I |
| PTPRZ1 | D | NS | R |
| PYGM | ND | NS | I |
| RYR2 | D | NS | I |
| SMAD4 | D | NS | R |
| TCF3 | D | NS | I |

| TGFBR2 TTN | D D | NS NS | I R |
| --- | --- | --- | --- |
|  |  | Serine Biosynthesis |  |
| DUSP26 | ND | R | R |
| PHGDH | D | NS | R |
| PSAT1 | D | NS | I |
|  |  | Sperm Motility |  |
| ATP1A4 | ND | NS | R |
| CACNA1H | D | NS | R |
| CATSPER1 | ND | NS | R |
| CNGA1 | ND | NS | I |
| CNGB1 | ND | NS | NS |
| GNAS | D | NS | NS |
| ITPR1 | D | NS | I |
| ITPR3 | D | NS | I |
| PDE1C | D | NS | I |
| PLA2G1B | ND | NS | I |
| PLA2G3 | ND | NS | I |
| PLA2G7 | D | NS | I |
| PLA2R1 | ND | NS | NS |
| PLCB1 | D | R | R |
| PRKAG2 | D | NS | I |
| PRKCA | D | NS | I |
| PRKCQ | D | NS | I |
| PTK2B | D | NS | I |
| UVA-Induced MAPK Signaling | | | |
| ART1 | ND | NS | NS |
| EGFR | D | I | I |
| HRAS | D | NS | R |
| MAPK10 | D | NS | I |
| PARP1 | D | NS | R |
| PARP11 | D | R | R |
| PARP4 | D | I | I |
| PARP9 | D | NS | I |
| PIK3C2A | D | NS | NS |
| PIK3C2B | D | NS | I |
| PIK3R1 | D | NS | NS |
| PIK3R2 | ND | NS | NS |
| PLCB1 | D | R | R |
| PRKCA | D | NS | I |
| RPS6KA2 | D | NS | I |
| SMPD3 | ND | NS | R |
| STAT1 | D | NS | NS |
| TIPARP | D | I | I |

Wnt/Ca+ pathway

CREBBP D NS NS

| PLCE1 | D | NS | NS |
| --- | --- | --- | --- |
| ROR1 | D | NS | NS |

D=Detected; ND=Not Detected (CPM<3) I=Increased; R=Reduced; NS=No Significant Change
