# Supplementary material for: Exome sequencing identifies gene variants and networks associated with extreme respiratory outcomes following preterm birth
Source: BMC Genet. 2018 Oct 20;19:94. doi: 10.1186/s12863-018-0679-7 (PMC6195962; doi:10.1186/s12863-018-0679-7)
Supplement: Supplementary file 9 — Table S9. Significant canonical pathways represented by unique variants in “affected” subjects from Li et al......... (DOCX 14 kb) [file 12863_2018_679_MOESM9_ESM.docx]

Supplemental Table 9. Significant canonical pathways represented by unique variants in “affected” subjects from Li et. al.

| **Canonical Pathways** | **-log(p- value)** | **z-score** | **Molecules** |
| --- | --- | --- | --- |
| Caveolar-mediated Endocytosis Signaling | 3.14 | DNP | ITGA9,ITGA2,COPB1,ITGB6,MAP3K2 |
| Tetrahydrofolate Salvage from 5,10-methenyltetrahydrofolate | 3.01 | DNP | MTHFD1L,MTHFD2L |
| Cdc42 Signaling | 2.83 | 2.236 | DIAPH1,RALA,LLGL1,ITGA2,LIMK2,APC,ITK |
| Histidine Degradation III | 2.57 | DNP | MTHFD1L,MTHFD2L |
| Folate Transformations I nNOS Signaling in Skeletal | 2.47 | DNP | MTHFD1L,MTHFD2L |
| Muscle Cells  Hepatic Fibrosis / Hepatic Stellate Cell Activation | 2.02  1.99 | DNP  DNP | CHRNA1,RYR2  COL5A2,FN1,COL6A3,COL12A1,KDR,TNFRSF11B |
| Factors Promoting Cardiogenesis in Vertebrates | 1.87 | DNP | TCF4,TGFBR3,LRP6,APC |
| Epithelial Adherens Junction Signaling | 1.8 | DNP | TCF4,LMO7,TGFBR3,JUP,APC |
| Paxillin Signaling | 1.73 | DNP | ITGA9,ITGA2,TLN1,ITGB6 |
| Polyamine Regulation in Colon Cancer | 1.7 | DNP | TCF4,APC |
| Actin Cytoskeleton Signaling Thiosulfate Disproportionation III (Rhodanese) | 1.66  1.53 | 0.816  DNP | DIAPH1,FN1,ITGA2,TLN1,LIMK2,APC  MOCS3 |
| Molecular Mechanisms of Cancer | 1.51 | DNP | ADCY9,TCF4,RALA,LRP6,ITGA2,ATR,GNA14,APC |
| PTEN Signaling | 1.51 | 1.000 | SYNJ2,TGFBR3,ITGA2,KDR |
| Sertoli Cell-Sertoli Cell Junction Signaling | 1.48 | DNP | SPTBN1,TGFBR3,ITGA2,JUP,MAP3K2 |
| Arginine Degradation I (Arginase Pathway) | 1.4 | DNP | ARG2 |
| Molybdenum Cofactor Biosynthesis | 1.4 | DNP | MOCS3 |
| Estrogen Receptor Signaling Role of Wnt/GSK-3β Signaling in the Pathogenesis of Influenza | 1.4  1.39 | DNP  DNP | NCOA2,GTF2E1,TRRAP,NCOR1  TCF4,NCOA2,APC |
| Cardiac β-adrenergic Signaling Inhibition of Angiogenesis by TSP1 | 1.35  1.35 | DNP  DNP | ADCY9,RYR2,SLC8A3,APEX1  SDC2,KDR |

DNP=Direction Not Predicted
