# Supplementary material for: Exome sequencing identifies gene variants and networks associated with extreme respiratory outcomes following preterm birth
Source: BMC Genet. 2018 Oct 20;19:94. doi: 10.1186/s12863-018-0679-7 (PMC6195962; doi:10.1186/s12863-018-0679-7)
Supplement: Supplementary file 10 — Table S10. Significant canonical pathways represented by unique variants in “unaffected” subjects from Li et al. (DOCX 22 kb) [file 12863_2018_679_MOESM10_ESM.docx]

Supplemental Table 10. Significant canonical pathways represented by unique variants in “unaffected” subjects from Li et. al.

**Canonical Pathways**

Amyotrophic Lateral

**-log(p-**

**value) z-score Molecules**

Sclerosis Signaling 2.64 DNP HECW1,GDNF,APAF1,GRIN3A,CAPN3

nNOS Signaling in

Skeletal Muscle Cells 2.33 DNP DAG1,CACNA2D3,CAPN3 GABA Receptor

Signaling 2.1 DNP GABRQ,ADCY8,GABRA2,CACNA2D3

Triacylglycerol

Degradation 2.02 DNP PPME1,LIPG,NDST2

Protein Kinase A

Signaling 1.73 DNP Synaptic Long Term

AKAP13,PTPN23,PTPRQ,CDC23,PPP1R3A,ADCY8,PTPRA,PL CH1

Potentiation 1.73 DNP PPP1R3A,ADCY8,PLCH1,GRIN3A HIPPO signaling 1.45 DNP PPP1R3A,BTRC,LATS2

tRNA Charging 1.39 DNP RARS2,LARS2

Dopamine-DARPP32 Feedback in cAMP

Signaling 1.33 DNP PPP1R3A,ADCY8,PLCH1,GRIN3A

Lactose Degradation III 1.31 DNP GLB1

DNP=Direction Not Predicted
